# Global estimates of pregnancies at risk of *Plasmodium falciparum* and *Plasmodium vivax* infection in 2020 and changes in risk patterns since 2000

**DOI:** 10.1371/journal.pgph.0001061

**Published:** 2022-11-09

**Authors:** Georgia R. Gore-Langton, Jorge Cano, Hope Simpson, Andrew Tatem, Natalia Tejedor-Garavito, Adelle Wigley, Alessandra Carioli, Peter Gething, Daniel J. Weiss, Daniel Chandramohan, Patrick G. T. Walker, Matthew E. Cairns, R. Matthew Chico

**Affiliations:** 1 Department of Disease Control, Faculty of Infectious and Tropical Diseases, London School of Hygiene & Tropical Medicine, London, United Kingdom; 2 Expanded Special Project for Elimination of Neglected Tropical Diseases, WHO Regional Office for Africa, Brazzaville, Democratic Republic of the Congo; 3 WorldPop, Geography and Environmental Science, University of Southampton, Southampton, United Kingdom; 4 Malaria Atlas Project, Telethon Kids Institute, Perth Children’s Hospital, Nedlands, Australia; 5 Faculty of Health Sciences, Curtin University, Bentley, Australia; 6 Faculty of Medicine, School of Public Health, Imperial College London, London, United Kingdom; 7 Department of Infectious Disease Epidemiology, Faculty of Epidemiology and Population Health, London School of Hygiene & Tropical Medicine, London, United Kingdom; Centre hospitalier de Cayenne, FRANCE

## Abstract

**Background:**

Women are at risk of severe adverse pregnancy outcomes attributable to *Plasmodium spp*. infection in malaria-endemic areas. Malaria control efforts since 2000 have aimed to reduce this burden of disease.

**Methods:**

We used data from the Malaria Atlas Project and WorldPop to calculate global pregnancies at-risk of *Plasmodium spp*. infection. We categorised pregnancies as occurring in areas of stable and unstable *P*. *falciparum* and *P*. *vivax* transmission. We further stratified stable endemicity as hypo-endemic, meso-endemic, hyper-endemic, or holo-endemic, and estimated pregnancies at risk in 2000, 2005, 2010, 2015, 2017, and 2020.

**Findings:**

In 2020, globally 120.4M pregnancies were at risk of *P*. *falciparum*, two-thirds (81.0M, 67.3%) were in areas of stable transmission; 85 2M pregnancies were at risk of *P*. *vivax*, 93.9% (80.0M) were in areas of stable transmission. An estimated 64.6M pregnancies were in areas with both *P*. *falciparum* and *P*. *vivax* transmission. The number of pregnancies at risk of each of *P*. *falciparum* and *P*. *vivax* worldwide decreased between 2000 and 2020, with the exception of sub-Saharan Africa, where the total number of pregnancies at risk of *P*. *falciparum* increased from 37 3M in 2000 to 52 4M in 2020.

**Interpretation:**

Historic investments in malaria control have reduced the number of women at risk of malaria in pregnancy in all endemic regions except sub-Saharan Africa. Population growth in Africa has outpaced reductions in malaria prevalence. Interventions that reduce the risk of malaria in pregnancy are needed as much today as ever.

## Introduction

### Plasmodium falciparum

*Plasmodium falciparum* infection during pregnancy increases the risk of spontaneous abortion [1], stillbirth [1], preterm delivery [2], low birthweight [3], small for gestational age [4], maternal and newborn anaemia [5], and maternal death [2, 4, 6]. In areas of high *P*. *falciparum* transmission, pregnant women develop partial immunity to placental infection through repeated malaria exposure over successive pregnancies [7–10]. The adverse effects of *P*.*falciparum* are largely due to the sequestration of the parasite within the placenta and are particularly seen in women who have not been exposed to *P*. *falciparum* in any previous pregnancy. The density and frequency of infection and the risk of associated adverse pregnancy outcomes are greatest among primigravidae, decreasing with each subsequent pregnancy.

The vast majority of global *P*. *falciparum* cases are in sub-Saharan African, whereas lower transmission persists across parts of South America and Asia [11, 12]. To protect pregnant women against the adverse consequences of malaria infection where transmission is moderate to high, the World Health Organization (WHO) recommends antenatal use of long-lasting insecticide-treated nets, active case management during pregnancy, and the provision of intermittent preventative treatment (IPTp) with sulfadoxine-pyrimethamine (SP) during scheduled antenatal care (ANC) visits from the second trimester to delivery [13]. Where *P*. *falciparum* transmission is low or unstable, there are no specific WHO prevention recommendations.

### Plasmodium vivax

*Plasmodium vivax* is also associated with stillbirth [1], preterm delivery [14], low birthweight [15, 16], and anaemia [16–18], but infection during pregnancy is considered less severe than *P*. *falciparum* in part because *P*. *vivax* infects only reticulocytes which limits parasite densities [18]. However, prevention and management of *P*. *vivax* infection can be challenging due to recrudescent and relapsing infections. Primaquine is used to treat *P*. vivax and is capable of eliminating liver-stage hypnozoites, but is contraindicated in pregnancy because of the potential to induce haemolysis and blood disorders in the fetus. Radical cure in pregnancy is not recommended until after delivery [13, 18]. Moreover, all gravidae infected with *P*. *vivax* are equally vulnerable to having low birthweight newborns [18]. Most *P*. *vivax* cases are in geographic areas of the WHO Regional Offices for South-East Asia and Eastern Mediterranean [19] where there are currently no WHO recommendations specifically for preventing these infections in pregnancy.

Global estimates of pregnancies at risk of malaria have not been generated since 2007 [20]. The most recent regional estimates were for 2020 and were specific to *P*. *falciparum* in sub-Saharan Africa [11]. Since 2007 there have been decreases in the prevalence of *P*. *falciparum* [12] and *P*. *vivax* [19], as well as global fertility rates [21, 22], although absolute populations have grown at regional and global levels. To support the data needs of policymakers and programme managers who are responsible for ongoing malaria control and elimination efforts in their advocacy for resources for malaria in pregnancy interventions and research, we have generated contemporary estimates of pregnancies at risk of *P*. *falciparum* and *P*. *vivax* stratified by transmission intensity.

## Methods

### Malaria estimates

The rate of *P*. *falciparum* infection among 2 to 10 year olds, as generated by the Malaria Atlas Project (MAP), has been used as a proxy for malaria among pregnant women [23, 24]. We obtained global estimates of mean *P*. *falciparum* and *P*. *vivax* parasite rates (all ages) from MAP as raster datasets at 5km^2^ resolution. These estimates incorporate prevalence data from household surveys, case data, and socio-economic and environmental data to produce smoothed, global estimates of malaria parasite rate (hereafter referred to as prevalence) [12, 25]. For our 2020 estimated pregnancies at risk of malaria infection, we calculated the central, lower, and upper (mean, 2 5^th^ and 97 5^th^ percentile, respectively) prevalence estimate for every 5km^2^ globally from 100 realisations of the underlying joint posterior prevalence of *P*. *falciparum* and *P*. *vivax*. For years 2000, 2005, 2010, 2015, and 2017 we used mean prevalence estimates as generated by MAP.

### Pregnancy estimates

We obtained global estimates of total pregnancies at 1km^2^ resolution from WorldPop. These data are based on age and sex structures of global population at 1km^2^ to estimate the number of children under one year of age. An extrapolation factor is then applied to correct for child mortality and to estimate the number of livebirths, which is then standardised to the United Nations total national number of livebirths [26–28]. Country specific numbers of stillbirths, miscarriages, and abortions are incorporated to estimate the ratio of livebirths to pregnancies [29]. yielding numbers of pregnancies per 1km^2^ for all African, Asian, and Latin American and Caribbean countries.

### Pregnancies at risk of malaria

We aggregated pregnancy estimates to the same resolution as MAP estimates (5km^2^) for all malaria-endemic countries and matched these to the corresponding year of malaria prevalence. Pregnancy-weighted malaria prevalence was averaged at the smallest administrative level of each country. For 2020, we classified pregnancies as being at risk of stable (≥0 01%, i.e. ≥ one case among 10,000 people per annum) or unstable (>0% and <0 01%) transmission based on the central, lower, and upper estimates of malaria prevalence [20]. We quantified overall pregnancies at risk by regions of the Sustainable Development Goals (SDG) and country, and by stable or unstable transmission, rounding the number of pregnancies at risk to the nearest hundred. We considered pregnant women resident in areas with both *P*. *falciparum* and *P*. *vivax*, stable or unstable, at risk of mixed infections. We further categorised stable malaria endemicity as hypo-endemic (0 01 to 10% inclusive), meso-endemic (11 to 50% inclusive), hyper-endemic (51 to 75 inclusive %) and holo-endemic (>75%) and summed pregnancy estimates by categorical endemicity for years 2000, 2005, 2010, 2015, 2017, and 2020 [30]. Finally, to investigate the impact of malaria control efforts over the past two decades, we calculated the hypothetical number of pregnancies at risk of each level of *P*. *falciparum* endemicity using WorldPop pregnancy estimates for 2020 and MAP prevalence estimates for 2000 and assumed that the number of pregnancies in 2020 no longer at risk of infection, or at lower risk of infection, were attributable to antimalarial interventions since 2000.

### Structure of results section

We first present 2020 global results for *P*. *falciparum* followed by *P*. *vivax*. We then report the proportion of at risk pregnancies by *P*. *falciparum* prevalence for all countries in sub-Saharan Africa and for *P*. *vivax* for countries where more than 10% of at risk pregnancies are in areas of stable transmission. We subsequently present pregnancies at risk of unstable/hypo-/meso-/hyper-/holo-endemic malaria for 2000, 2005, 2010, 2015, 2017, 2020 by malaria species. Finally, we report the change in the number of pregnancies at risk of *P*. *falciparum* in 2020 compared to the counterfactual number of pregnancies that would have been at risk had malaria prevalence remained at 2000 levels.

### Role of the funding source

The study sponsors had no role in any stage of data analysis or manuscript development.

## Results

### *Plasmodium falciparum*: Stable versus unstable transmission

In 2020, 120 4M pregnancies were at risk of *P*. *falciparum* transmission worldwide (Table 1). Over half of these (81.0M, 67.25%) were in areas of stable (≥0 01%) transmission. The majority of global pregnancies at risk of stable transmission were in sub-Saharan Africa, 52 4M (Table 1). Considering SDG regions, Central and Southern Asia had the second largest number of pregnancies at risk of stable *P*. *falciparum*, 19.4M, making up 40 53% of total pregnancies at risk in the region, the remaining pregnancies were at risk of unstable *P*. *falciparum* (Table 1). The provision of IPTp is WHO policy in all areas of meso-endemic (11 to 50%) or higher transmission within sub-Saharan Africa [31]. An estimated 34 8M pregnancies met these criteria in 2020 (S1 Table), with a further 93,100 pregnancies exposed to similar levels of transmission outside of sub-Saharan Africa.

**Table 1:** Pregnancies at risk of *Plasmodium falciparum* and *Plasmodium vivax* in 2020 by regions of the Sustainable Development Goals disaggregated by stable and unstable transmission.

	***Plasmodium falciparum***				
		**Stable transmission (> = 0.01%)**		**Unstable transmission (<0.01%)**	
**SDG region**	**Total pregnancies at risk**	**Pregnancies at risk** **(2.5**^**th**^ **to 97.5**^**th**^ **percentile)**	**% of total pregnancies** **(2.5**^**th**^ **to 97.5**^**th**^ **percentile)**	**Pregnancies at risk** **(2.5**^**th**^ **to 97.5**^**th**^ **percentile)**	**% of total pregnancies** **(2.5**^**th**^ **to 97.5**^**th**^ **percentile)**
**Northern Africa & Western Asia**	4,131,700	3,243,300 (2,714,300–3,259,500)	78.50 (65.69–78.89)	888,400 (872,200–1,417,400)	21.50 (21.11–34.31)
**Sub-Saharan Africa**	52,395,400	52,320,300 (52,132,600–52,352,900)	99.86 (99.50–99.92)	75,100 (42,600–262,800)	0.14 (0.08–0.50)
**Central & Southern Asia**	47,849,200	19,393,500 (4,805,600–32,302,300)	40.53 (10.04–67.51)	28,455,700 (15,546,900–42,030,500)	59.47 (32.49–87.84)
**Eastern & South-Eastern Asia**	10,090,300	3,968,300 (211,500–6,029,700)	39.33 (2.10–59.76)	6,122,000 (4,060,600–9,878,800)	60.67 (40.24–97.90)
**Latin America & the Caribbean**	5,979,000	2,075,700 (89,000–2,620,200)	34.72 (1.49–43.82)	3,903,300 (3,176,600–5,453,100)	65.28 (53.13–91.20)
**Global Total**	120,445,600	81,001,100 (59,953,000–96,564,600)	67.25 (49.78–80.17)	39,444,500 (23,698,900–59,042,600)	32.75 (19.68–49.02)
	***Plasmodium vivax***				
		**Stable transmission (> = 0.01%)**		**Unstable transmission (<0.01%)**	
	**Total pregnancies at risk**	**Pregnancies at risk** **(2.5**^**th**^ **to 97.5**^**th**^ **percentile)**	**% of total pregnancies** **(2.5**^**th**^ **to 97.5**^**th**^ **percentile)**	**Pregnancies at risk** **(2.5**^**th**^ **to 97.5**^**th**^ **percentile)**	**% of total pregnancies** **(2.5**^**th**^ **to 97.5**^**th**^ **percentile)**
**Northern Africa & Western Asia**	3,077,900	2,776,300 (2,712,200–2,822,800)	90.20 (88.12–91.71)	301,600 (255,100–365,700)	9.80 (8.29–11.88)
**Sub-Saharan Africa**	6,995,700	6,985,300 (6,985,300–6,985,300)	99.85 (99.85–99.85)	10,400 (10,400–10,400)	0.15 (0.15–0.15)
**Central & Southern Asia**	54,564,600	51,748,000 (51,175,600–52,601,500)	94.84 (93.79–96.40)	2,816,600 (1,963,100–3,389,000)	5.16 (3.60–6.21)
**Eastern & South-Eastern Asia**	12,900,200	12,279,300 (9,995,100–12,490,600)	95.19 (77.48–96.82)	620,900 (409,600–2,905,100)	4.81 (3.18–22.52)
**Latin America & the Caribbean**	7,649,400	6,182,900 (4,386,000–6,539,600)	80.83 (57.34–85.49)	1,466,500 (1,109,800–3,263,400)	19.17 (14.51–42.66)
**Global Total**	85,187,800	79,971,800 (75,254,200–81,439,800)	93.88 (88.34–95.60)	5,216,000 (3,748,000–9,933,600)	6.12 (4.40–11.66)

In 2020, 39.4M pregnancies globally were at risk of unstable *P*. *falciparum* transmission (Table 1).

With the exceptions of sub-Saharan Africa and Northern Africa and Western Asia, the majority of at risk pregnancies across SDG regions were in areas of unstable *P*. *falciparum* transmission. Central and Southern Asia had the largest number of pregnancies at risk of unstable *P*. *falciparum* (28 5M) making up 59.47% of the region’s at risk pregnancies. Eastern and South-Eastern Asia had the second highest number of pregnancies at risk of unstable *P*. *falciparum* transmission, 6.1M, accounting for 60 7% of at risk pregnancies in the region (Table 1).

## Countries and regions of sub-Saharan Africa

### Population at risk

**Fig 1:**
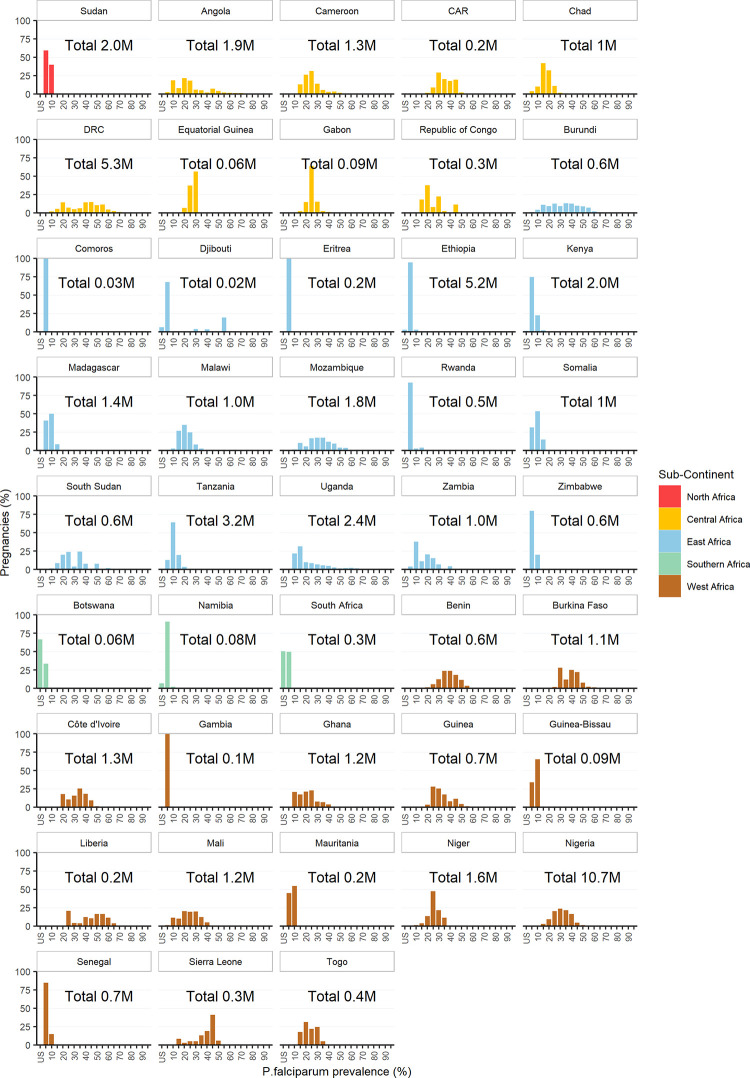
Proportion and total number of pregnancies at risk of *Plasmodium falciparum* in Africa in 2020 by country and prevalence (%); US = unstable (0> and <0 01%).

In East Africa, Ethiopia had the most pregnancies at risk of *P*. *falciparum*, 5 2M, of which 99 7% were in areas of stable transmission (Fig 1), although prevalence was relatively low (mean 2 3% IQR: 0 1 to 5 6) (S2 Table). In West Africa, Nigeria had the most pregnancies at risk, 10 7M, all of which were in areas of stable *P*. *falciparum* transmission where the mean prevalence was 29 9% (IQR: 15 8 to 43 7). In Central Africa, the Democratic Republic of Congo (DRC) had the most pregnancies at risk of *P*. *falciparum* infection, 5 3M, all in areas of stable transmission where the mean prevalence was 39 7% (IQR: 8 9 to 68 8) (Fig 1 and S2 Table).

### Prevalence of at risk population

Liberia was the country with the highest mean prevalence of *P*. *falciparum* within sub-Saharan Africa, 49 3% (IQR: 31 8 to 62 9) in areas of stable transmission and 214,600 pregnancies at risk, followed by DRC, as described above, and Sierra Leone, mean prevalence 39 2% (IQR: 22 7 to 46.0) and 328,800 pregnancies at risk. The top five countries globally with most pregnancies at risk of stable *P*. *falciparum* transmission were, in descending order: India, Nigeria, DRC, Ethiopia, and Pakistan; mean prevalence was highest in DRC and Nigeria. The top five countries with the most pregnancies in areas of unstable *P*. *falciparum* transmission (<0 01% prevalence) were, in descending order: India, Indonesia, Brazil, Afghanistan, and Vietnam.

### High burden high impact countries

Eleven countries are considered “High burden to high impact countries” that account for approximately 70% of the global *P*. *falciparum* [32]. Among these, India had the most pregnancies at risk of *P*. *falciparum* (37 5M), of which 38.0% were at risk of stable but low transmission (mean: 0 23%; IQR: 0 01 to 1.61) (S1 Fig and S2 Table). Among this group of countries Nigeria and DRC had the next highest numbers of pregnancies at risk as detailed above (S1 and S2 Figs).

### Plasmodium vivax

Worldwide, 85 2M pregnancies were at risk of *P*. *vivax*, of which 80 0M (93 9%) were in areas of stable transmission. The majority of pregnancies at risk of stable *P*. *vivax* transmission were in Central and Southern Asia (51 7M), accounting for 94 8% of all pregnancies at risk of *P*. *vivax* in the region. Globally, 5.2M pregnancies were in areas of unstable *P*. *vivax* transmission. A total of 7 0M pregnancies within sub-Saharan Africa were at risk of *P*. *vivax* (99.9% of which in areas of stable transmission).

**Fig 2:**
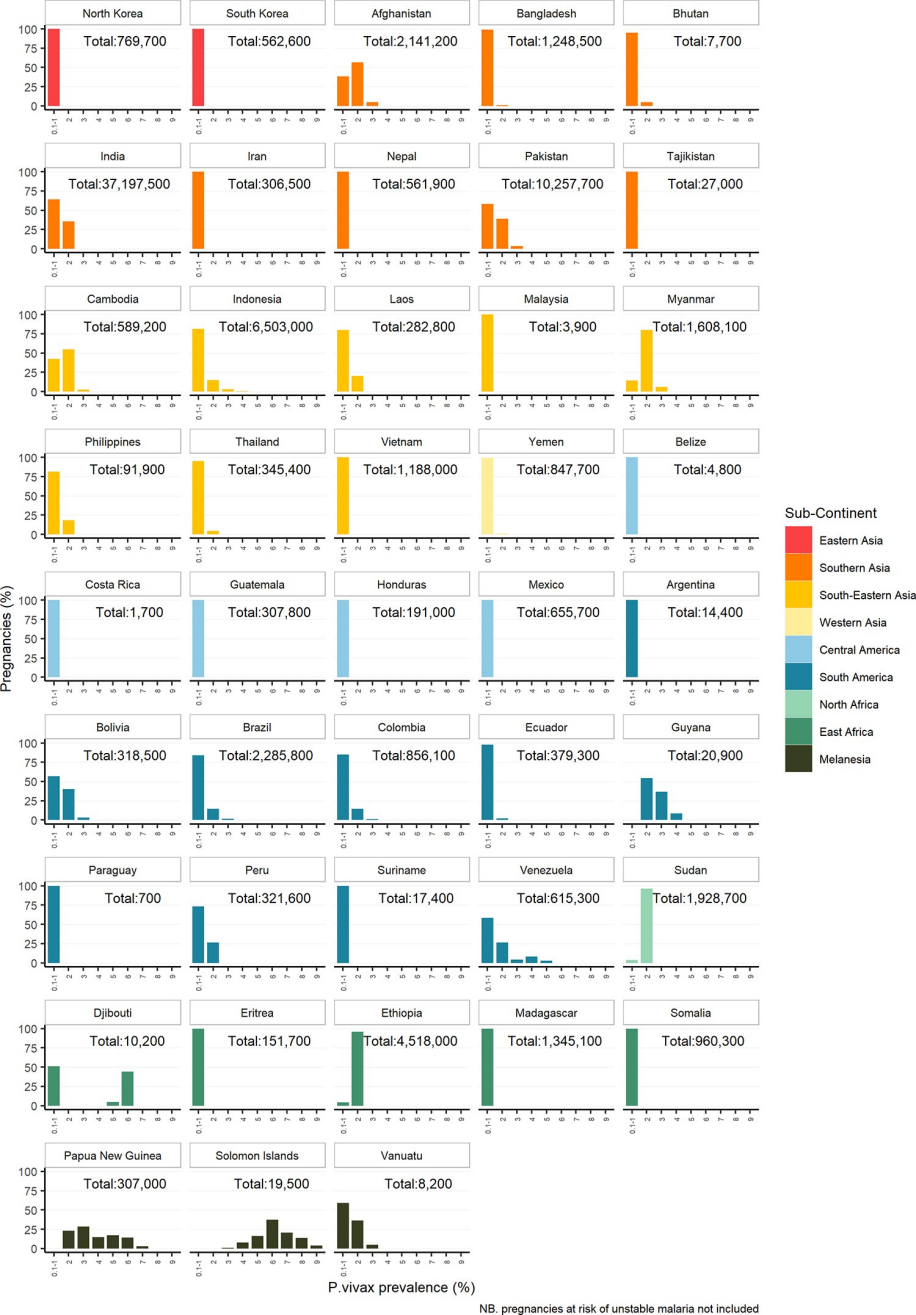
Proportion and total number of pregnancies at risk of stable *Plasmodium vivax* in 2020 in countries where >10% of total at-risk pregnancies are at-risk of stable transmission.

The proportion of pregnancies at risk of stable *P*. *vivax* by prevalence is shown in Fig 2 for countries where more than 10% of the pregnancies at risk of *P*. *vivax* were in areas of stable transmission. India had the largest number of pregnancies at risk of *P*.*vivax*, 38 3M, 97.2% of these (37.2M) were in areas of stable transmission where mean prevalence was 0 4%, IQR: 0 06 to 0 97, followed by Pakistan with 10.3M pregnancies all at risk of stable transmission, mean prevalence 0.76, IQR: 0.07 to 1.65). Indonesia had the third largest number of pregnancies at risk of *P*.*vivax*; in total 6.7M pregnancies were at risk, 96.8% (6.5M) of these were in areas of stable transmission where mean prevalence was 0.55, IQR: 0.03 to 2.24).

### Mixed infections

Worldwide, 64.6M pregnancies in 2020 were resident in areas of both *P*. *falciparum* and *P*. *vivax* transmission, and therefore at risk of mixed infections. The majority of these (41 5M, 64 2%) were within Central & Southern Asia (S3 Table and S3 Fig).

### Changes from 2000 to 2020

**Fig 3:**
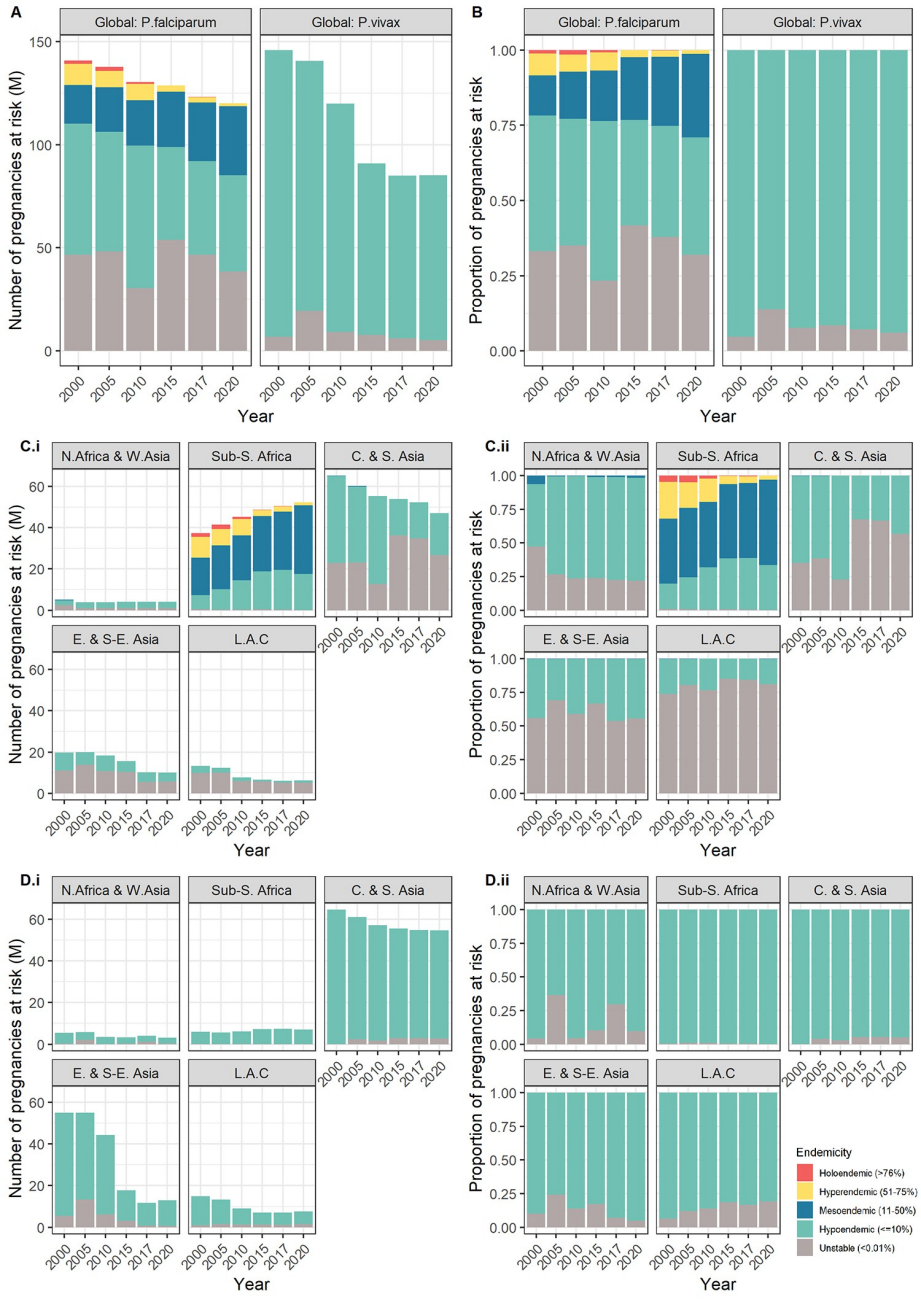
Number (A) and proportion (B) of pregnancies at risk of *Plasmodium falciparum* and *Plasmodium vivax* globally from 2000 to 2020, and number (C.i) and proportion (C.ii) of pregnancies at risk of *Plasmodium falciparum* and *Plasmodium vivax* (D.i and D.ii) from 2000 to 2020 by SDG region.

**Fig 4:**
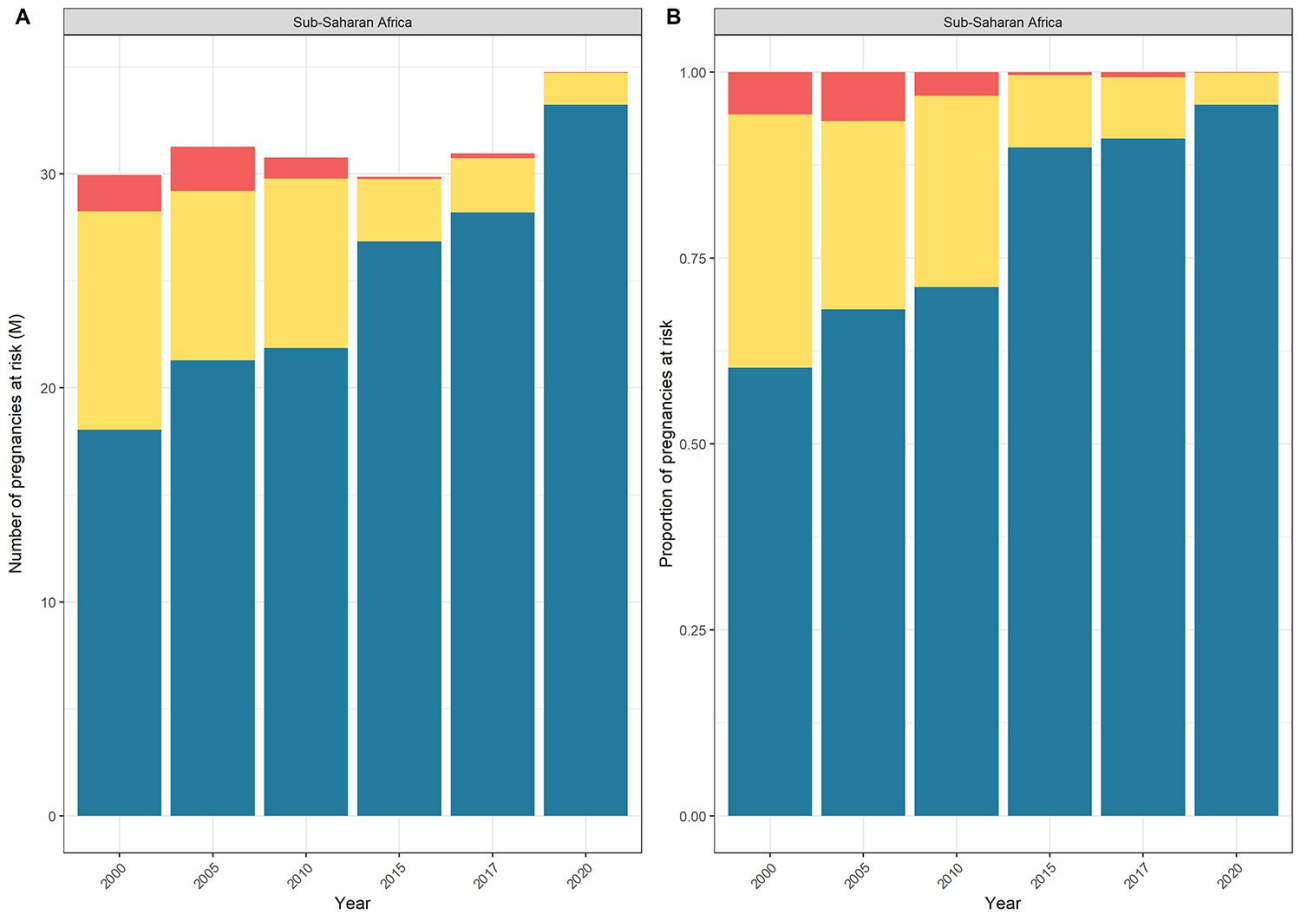
Number (A) and proportion (B) of pregnancies at risk of meso-endemic or higher (≥ 11%) *Plasmodium falciparum* transmission in sub-Saharan Africa, 2000 to 2020.

The total number of pregnancies at risk of *P*. *falciparum* or *P*. *vivax* globally decreased between 2000 and 2020 (Fig 3A and Fig 3B). Despite malaria prevalence having decreased overall in sub-Saharan Africa since 2000, and the number and proportion of pregnancies exposed to hyper- (51 to75%) or holo-endemic (>75%) malaria decreasing, the total number of pregnancies at risk of *P*. *falciparum* increased year-on-year between 2000 and 2020 in sub-Saharan Africa from 37 3M to 52 4M (Fig 3C and S4 Table), with nearly all of these pregnancies in areas of stable transmission. This rise was driven by an increase in the number of pregnancies at risk of lower levels of transmission (hypo- [0.01 to 10%] and meso-endemic [11 to 50%]) (Fig 3C and S4 Fig). There were 10.4M fewer pregnancies in areas of hyper- (51 to 75%) or holo-endemic (>75%) *P*. *falciparum* in 2020 than in 2000, and 25.7M more pregnancies in areas of the lower levels of hypo-endemic (0.1 to 10%) or meso-endemic (11 to 50%) *P*. *falciparum* in 2020 than in 2000 (S4 Fig). The number of pregnancies residing in areas of meso-endemic (11 to 50%) transmission or higher within sub-Saharan Africa, and therefore eligible for IPTp, increased from 30 0M in 2000 to 34 8M in 2020, with the steepest rise between 2017 and 2020 (Fig 4 and S1 Table). The number and percentage of pregnancies by *P*. *falciparum* endemicity is shown by year for African countries in S5 and S6 Figs.

Across Asia, pregnancies at risk of *P*. *falciparum* decreased from 2000 to 2020, as did the proportion of at risk pregnancies in areas of higher transmission levels (Fig 3C). Global pregnancies at risk of *P*. *vivax* decreased between 2000 and 2020, with a slight increase between 2017 and 2020. The number and proportion of pregnancies by *P*. *vivax* endemicity is shown by year for Asian countries in S7 and S8 Figs. In sub-Saharan Africa, numbers at risk of *P*. *vivax* increased from 5.9M in 2000 to 7.0M in 2020 (Fig 3D).

The total number of pregnancies (including those not at risk of malaria) in countries with endemic *P*. *falciparum* has increased between 2000 and 2020 while the proportion of pregnancies at risk of *P*. *falciparum* (stable or unstable) has decreased; 75.5% of all pregnancies in *P*. *falciparum* endemic countries in 2000 were in areas of malaria transmission, reducing to 62.8% in 2020 (S9 Fig and S4 Table). The total number of pregnancies in countries endemic for *P*. *vivax* has decreased between 2000 and 2020, as did the proportion of total pregnancies at risk of *P*. *vivax;* 95.6% of all pregnancies within countries with endemic *P*. *vivax* were at risk of *P*. *vivax* in 2000, reducing to 59.5% in 2020. In sub-Saharan Africa, there has been a year-on-year increase in both the total number of pregnancies and the proportion of total pregnancies at risk of each of *P*. *falciparum* and *P*. *vivax*, applicable to the six African countries for which MAP generate *P*. *vivax* transmission (Ethiopia, Eritrea, Djibouti, Somalia, Sudan, and Madagascar) (S9 Fig and S4 Table).

### Counterfactuals

In sub-Saharan Africa there were just over 2.4 million fewer pregnancies in 2020 in areas of holo-endemic (>75%) *P*. *falciparum* transmission and 13 4M fewer pregnancies in areas of hyper-endemic (51 to 75%) transmission than there would have been had prevalence remained at 2000 levels but pregnancies continued to increase to 2020 levels (S10 Fig). Thus, more pregnant women were resident in areas where transmission is less intense. Specifically, 16 1M more sub-Saharan pregnant women were living in hypo-endemic (0.1 to 10%) and meso-endemic (11 to 50%) areas in 2020 compared to 2000. In Central and Southern Asia, there were 13.5M pregnancies in 2020 in areas of no *P*. *falciparum* transmission which would have been at risk in 2000, and 5.1M more pregnancies in areas of unstable transmission (>0 and <0.01%), reflecting the reduction of 18 6M pregnancies in areas of hypo-endemic (0.1 to 10%) and meso-endemic (11 to 50%) transmission (S9 Fig).

## Discussion

We present contemporary global estimates of pregnancies at risk of *P*. *falciparum* and *P*. *vivax* and examine changes in risk by categorical endemicity since 2000. Despite decreases in *P*. *falciparum* and *P*. *vivax* prevalence, and decreases in the global number of pregnancies at risk of malaria, the number of pregnancies at risk of moderate to high *P*. *falciparum* transmission in sub-Saharan Africa i.e. women requiring preventative intervention, has remained relatively unchanged, increasing between 2017 and 2020. The scale up of antimalarial interventions for pregnant women throughout the antenatal period is needed today as much as ever and, indeed, should precede conception given that an estimated 70% of infections among primigravidae are acquired before pregnancy [9]. Redoubled efforts are needed to reduce malaria exposure with long-lasting insecticide nets and prompt case management among women of child bearing age.

Our analysis illustrates the challenges of reducing the number and proportion of pregnant women at risk of malaria infection in sub-Saharan Africa, where fertility rates are decreasing, but remain high (S10 Fig), and population growth is forecasted up to and after 2100 [33, 34]. While not an antimalarial intervention, efforts that strengthen family planning services in malaria-endemic countries contribute to maternal and child health as well as reduce the number of pregnancies at risk of malaria.

We have shown that malaria control efforts have led to a reduction in the number of pregnancies at risk of higher levels of *P*. *falciparum* transmission within sub-Saharan Africa, and a corresponding increase in the numbers at risk of lower levels of transmission. Across other regions, pregnancies at risk of hypo-endemic (>0.01 to 10%) transmission have decreased while the numbers in areas of unstable (<0.01%) or no *P*. *falciparum* transmission have increased. Approximately a third of all pregnancies at risk of *P*. *falciparum* worldwide were in areas of very low, unstable transmission where prevention recommendation are missing, and if they were to be present, would be operationally challenging to deploy and may be difficult to justify on a cost-effectiveness basis unless the value of malaria elimination is also taken into consideration. Nonetheless, this burden of disease is not insignificant given the very large number of pregnancies in consideration, the lower acquired levels of immunity in these transmission settings and the higher likelihood of infections evolving to clinical disease [35]. While pregnancies at risk of *P*. *vivax* are fewer in number and *P*. *vivax* commonly considered to be more benign than *P*. *falciparum*, research investment is urgently needed given the millions of pregnant women at risk of *P*. *vivax* infection for which there is no radical cure during pregnancy and increasingly strong evidence of its distribution across malaria-endemic Africa [36].

Women of all gravidities who reside in areas of unstable transmission are at risk of developing high-density placental infections with potential for adverse maternal and newborn health outcomes. The association between *P*. *falciparum* during pregnancy and stillbirth is stronger in areas of low to intermediate transmission than in areas of high transmission [1]. Access to rapid diagnosis and treatment of these uncommon but dangerous infections in areas of unstable transmission is important.

Within areas of stable transmission, malaria prevalence varied widely. The WHO recommends the provision of IPTp with sulfadoxine-pyrimethamine to pregnant women resident in areas of moderate to high *P*. *falciparum* transmission in Africa, an intervention that has potent anti-malarial and non-malarial effects on birthweight [31, 37] Decreases in *P*. *falciparum* transmission across the continent have been insufficient to translate into significant decreases in the number of pregnancies requiring IPTp, while IPTp coverage continues to lag [38].

Our estimates suggest there were 35.1M more pregnancies at risk of *P*. *falciparum* and 7.7M fewer pregnancies at risk of *P*. *vivax* in 2020 than were estimated by the previous global estimates for 2007 [20]. These 2007 estimates used MAP transmission limits, separating pregnancies by unstable or stable transmission. The more recent MAP prevalence estimates allowed us to stratify at risk pregnancies by prevalence in more detail than just stable/unstable, and the use of WorldPop estimates of numbers of pregnancies at 5^km^ resolution enable us to estimate number of pregnancies, and the level of malaria transmission within which they reside, at a much finer geographic resolution than was applied to 2007 estimates [20]. The World Malaria Report of 2021 estimated that 33 8M pregnancies were at risk of moderate to high *P*. *falciparum* transmission in sub-Saharan Africa in 2020 [11], one million fewer than our estimate for the same year.

Our analyses have limitations. In the absence of malaria prevalence estimates specifically for pregnant women, we have used *P*. *falciparum* prevalence estimates generated by MAP for 2 to 10 year-old children. Previous estimates of pregnancies at risk of malaria have used the same metric [20, 38], and literature suggests prevalence among children is representative of prevalence among pregnant women, especially among primigravidae and in areas of low transmission (<5%) [23]. In the case of *P*. *vivax*, we used data available from MAP which represents the prevalence across all age groups, the only available global *P*. *vivax* estimates. Averaging prevalence at the smallest administrative region of each country is pragmatic, however, it means that all pregnancies within an administrative region that has only hot-spots of transmission are classified as being at risk of malaria. As transmission decreases and becomes more localised, a more granular approach to estimating risk of malaria may become more appropriate. Both MAP and WorldPop estimates come with their own limitations and uncertainties. The uncertainty in 2020 MAP estimates is captured in the range between 2.5^th^, mean, and 97.5^th^ percentile prevalence. WorldPop estimates for pregnancies have been computed using the total number of children under one year of age to quantify the number of live births first and then pregnancies. This can result in ecological fallacies as demographic rates at national level are applied to sub-national levels, resulting in possible over- or under-estimation of pregnancies. Similar variability may occur when using defined administrative boundaries to summarise the pregnancies. This is known as the Modifiable Areal Unit Problem (MAUP), which describes how spatial summary measures are inherently influenced by the administrative boundaries that they are reported at [39]. In addition, our estimates of pregnancies at risk of malaria in 2020 do not reflect the seasonality of malaria transmission in some settings. Knowledge of local variation in transmission is important when planning prevention and treatment interventions in areas of highly seasonal transmission. Our estimates are also not stratified by gravidity and so do not allow for estimation of primigravid pregnancies within areas of *P*. *falciparum* transmission, i.e. those at greatest risk of adverse outcomes.

The Covid-19 pandemic has disrupted malaria control programmes and health systems in general and malaria prevalence and cases greatly increased in 2020 [40, 41]. Indeed, matching the reductions in malaria prevalence over the next two decades may be challenging and the total population at risk of malaria is projected to increase over the coming 50 years [42]. New tools are increasingly available for use in malaria control and elimination, specifically malaria vaccines could increasingly feature in reducing the burden of malaria in pregnancy, potentially in combination with malaria chemoprevention [43]. How these tools may be best deployed to safeguard pregnant women at risk of malaria infection by transmission intensity and malaria species will need to be evaluated.

## Supporting information

S1 Table Pregnancies within sub-Saharan Africa at risk of moderate or high (meso-endemic (11–50%) or higher transmission) *Plasmodium falciparum* by year. (DOCX)

S2 Table Total number of pregnancies at risk of malaria in 2020 and mean (2.5th to 97.5th percentile) prevalence by country and Plasmodium species, disaggregated by stable or unstable transmission. NB: where Mean (2.5th to 97.5th percentile) prevalence (%) is noted as “<0.001” all three measures of prevalence <0.001%.
(DOCX)

S3 Table Pregnancies at risk of *Plasmodium falciparum* and *Plasmodium vivax* in 2020 by regions of the Sustainable Development Goals. (DOCX)

S4 Table Number of pregnancies at risk of *Plasmodium falciparum* or *P*.*vivax* by SDG and year. (DOCX)

S1 Fig Number of pregnancies at risk of *Plasmodium falciparum* in 2020 in two High Burden High Impact Countries: Nigeria and India. (TIFF)

S2 Fig Number of pregnancies at risk of Plasmodium falciparum in 2020 in each High Burden High Impact Country (except Nigeria and India, see Fig 1). (TIFF)

S3 Fig Areas at risk of both *Plasmodium falciparum* and *Plasmodium vivax* in 2020. Note: Map made with Natural Earth. Free vector and raster map data @ naturalearthdata.com.
(TIFF)

S4 Fig Change in number of pregnancies at risk of *Plasmodium falciparum* in sub-Saharan Africa by categorical endemicity between 2000 and 2020 (M). (TIFF)

S5 Fig (TIFF)

S6 Fig Proportion of pregnancies at risk of *Plasmodium falciparum* within Africa by country from 2000 to 2020. (TIFF)

S7 Fig Number of pregnancies at risk of *Plasmodium vivax* within Asia by country from 2000 to 2020. (TIFF)

S8 Fig Proportion of pregnancies at risk of *Plasmodium vivax* within Asia by country from 2000 to 2020. (TIFF)

S9 Fig Number (A) and proportion (B) of pregnancies in countries of Plasmodium falciparum and *Plasmodium vivax* transmission globally from 2000 to 2020 disaggregated by risk of malaria or no risk of malaria, and number (C.i) and proportion (C.ii) of pregnancies by risk of *Plasmodium falciparum* and *Plasmodium vivax* (D.i and D.ii) from 2000 to 2020 by geographic region.
(TIFF)

S10 Fig Difference in pregnancies at risk of *Plasmodium falciparum*, by categorical endemicity, in 2020 compared to numbers expecting according to 2000 prevalence estimates and 2020 pregnancy estimates 20. (TIFF)

S11 Fig Proportion of births that are a woman’s first birth and total fertility rate per country in sub-Saharan Africa. (TIFF)
