# Supplementary material for: Global estimates of pregnancies at risk of Plasmodium falciparum and Plasmodium vivax infection in 2020 and changes in risk patterns since 2000
Source: PLOS Glob Public Health. 2022 Nov 9;2(11):e0001061. doi: 10.1371/journal.pgph.0001061 (PMC10022219; doi:10.1371/journal.pgph.0001061)
Supplement: S1 Table — (DOCX) [file pgph.0001061.s001.docx]

| **Year** | **Pregnancies at risk of moderate or high *P. falciparum* within SSA (M)** |
| --- | --- |
| **2000** | 29.95 |
| **2005** | 31.27 |
| **2010** | 30.76 |
| **2015** | 29.87 |
| **2017** | 30.96 |
| **2020** | 34.76 |
