# Supplementary material for: Global estimates of pregnancies at risk of Plasmodium falciparum and Plasmodium vivax infection in 2020 and changes in risk patterns since 2000
Source: PLOS Glob Public Health. 2022 Nov 9;2(11):e0001061. doi: 10.1371/journal.pgph.0001061 (PMC10022219; doi:10.1371/journal.pgph.0001061)
Supplement: S2 Table — NB: where Mean (2.5th to 97.5th percentile) prevalence (%) is noted as “<0.001” all three measures of prevalence <0.001%. (DOCX) [file pgph.0001061.s002.docx]

# *S2 Table:* Total number of pregnancies at risk of malaria in 2020 and mean (2.5^th^ to 97.5^th^ percentile) prevalence by country and *Plasmodium* species, disaggregated by stable or unstable transmission. NB: where mean (2.5th to 97.5th percentile) prevalence (%) is noted as “<0.001” all three measures of prevalence <0.001%

|  |  |  | **P.falciparum** | | | | | **P.vivax** | | | | |
| --- | --- | --- | --- | --- | --- | --- | --- | --- | --- | --- | --- | --- |
| **SDG region** | **Country** | **Transmission** | **Pregnancies at risk in country**  **(n)** | **Pregnancies at risk by transmission category (n)** | **Pregnancies at risk by transmission category (%)** | **Mean**  **(2.5^th^ to 97.5^th^ percentile) prevalence (%)** | **Pregnancies at risk in country (n)** | | **Pregnancies at risk by transmission category (n)** | **Pregnancies at risk by transmission category (%)** | **Mean**  **(2.5^th^ to 97.5^th^ percentile) prevalence (%)** |  |
| Northern Africa & Western Asia | Azerbaijan | Unstable (<0.01%) |  |  |  |  | 6,100 | | 6,100 | 100 | <0.001 |  |
|  | Iraq | Unstable (<0.01%) |  |  |  |  | 94,500 | | 94,500 | 100 | <0.001 |  |
|  | Saudi Arabia | Unstable (<0.01%) | 900,100 | 855,600 | 95.06 | <0.001 | 44,500 | | 44,500 | 100 | <0.001 |  |
|  | Saudi Arabia | Stable (>=0.01%) | 900,100 | 44,500 | 4.94 | 0.02(0.02-0.02) |  | |  |  |  |  |
|  | Sudan | Stable (>=0.01%) | 2,016,100 | 2,016,100 | 100 | 4.29(0.42-8.52) | 1,928,700 | | 1,928,700 | 100 | 0.76(0.55-0.97) |  |
|  | Turkey | Unstable (<0.01%) |  |  |  |  | 16,800 | | 16,800 | 100 | <0.001 |  |
|  | Yemen | Unstable (<0.01%) | 1,215,500 | 32,800 | 2.7 | 0(0-0.01) | 987,300 | | 139,600 | 14.14 | 0(0-0.01) |  |
|  | Yemen | Stable (>=0.01%) | 1,215,500 | 1,182,700 | 97.3 | 1.92(0.03-13.66) | 987,300 | | 847,700 | 85.86 | 0.16(0.01-0.42) |  |
| Sub-Saharan Africa | Angola | Stable (>=0.01%) | 1,875,800 | 1,875,800 | 100 | 34.48(5.87-70.23) |  | |  |  |  |  |
|  | Benin | Stable (>=0.01%) | 593,400 | 593,400 | 100 | 37.02(22.19-51.04) |  | |  |  |  |  |
|  | Botswana | Unstable (<0.01%) | 61,700 | 18,400 | 29.82 | 0(0-0.01) |  | |  |  |  |  |
|  | Botswana | Stable (>=0.01%) | 61,700 | 43,300 | 70.18 | 0.11(0.05-0.24) |  | |  |  |  |  |
|  | Burkina Faso | Stable (>=0.01%) | 1,106,900 | 1,106,900 | 100 | 37.86(24.96-53.29) |  | |  |  |  |  |
|  | Burundi | Stable (>=0.01%) | 617,800 | 617,800 | 100 | 32.3(6.19-55.31) |  | |  |  |  |  |
|  | Cameroon | Stable (>=0.01%) | 1,330,000 | 1,330,000 | 100 | 26.65(12.31-50.22) |  | |  |  |  |  |
|  | Central African Republic | Stable (>=0.01%) | 238,800 | 238,800 | 100 | 31.94(19.65-45.89) |  | |  |  |  |  |
|  | Chad | Stable (>=0.01%) | 998,300 | 998,300 | 100 | 15.03(7.05-24.95) |  | |  |  |  |  |
|  | Comoros | Stable (>=0.01%) | 29,300 | 29,300 | 100 | 0.62(0.61-0.63) |  | |  |  |  |  |
|  | Côte d'Ivoire | Stable (>=0.01%) | 1,292,200 | 1,292,200 | 100 | 32.06(17.04-48.39) |  | |  |  |  |  |
|  | Democratic Republic of the Congo | Stable (>=0.01%) | 5,281,100 | 5,281,100 | 100 | 39.74(8.85-68.8) |  | |  |  |  |  |
|  | Djibouti | Unstable (<0.01%) | 21,500 | 1,300 | 6.05 | 0.01(0.01-0.01) | 20,600 | | 10,400 | 50.49 | 0.01(0-0.01) |  |
| Sub-Saharan Africa | Djibouti | Stable (>=0.01%) | 21,500 | 20,200 | 93.95 | 28.57(0.38-54.57) | 20,600 | | 10,200 | 49.51 | 3.13(0.11-4.73) |  |
|  | Equatorial Guinea | Stable (>=0.01%) | 61,100 | 61,100 | 100 | 25.52(17.83-29.38) |  | |  |  |  |  |
|  | Eritrea | Stable (>=0.01%) | 152,800 | 152,800 | 100 | 0.98(0.56-1.74) | 151,700 | | 151,700 | 100 | 0.36(0.22-0.47) |  |
|  | Ethiopia | Unstable (<0.01%) | 5,169,500 | 16,200 | 0.31 | <0.001 |  | |  |  |  |  |
|  | Ethiopia | Stable (>=0.01%) | 5,169,500 | 5,153,300 | 99.69 | 2.32(0.14-5.58) | 4,518,000 | | 4,518,000 | 100 | 0.71(0.48-1) |  |
|  | Gabon | Stable (>=0.01%) | 88,500 | 88,500 | 100 | 22.42(15.01-34.69) |  | |  |  |  |  |
|  | Gambia | Stable (>=0.01%) | 115,200 | 115,200 | 100 | 3.24(2.62-4.58) |  | |  |  |  |  |
|  | Ghana | Stable (>=0.01%) | 1,197,000 | 1,197,000 | 100 | 20.67(9.66-37.48) |  | |  |  |  |  |
|  | Guinea | Stable (>=0.01%) | 651,000 | 651,000 | 100 | 32.63(19.35-53.69) |  | |  |  |  |  |
|  | Guinea-Bissau | Stable (>=0.01%) | 90,200 | 90,200 | 100 | 5.82(2.18-9.39) |  | |  |  |  |  |
|  | Kenya | Stable (>=0.01%) | 2,025,400 | 2,025,400 | 100 | 3.56(0.31-9.94) |  | |  |  |  |  |
|  | Liberia | Stable (>=0.01%) | 214,600 | 214,600 | 100 | 49.25(31.81-62.95) |  | |  |  |  |  |
|  | Madagascar | Stable (>=0.01%) | 1,376,600 | 1,376,600 | 100 | 6.27(1.91-11.12) | 1,345,100 | | 1,345,100 | 100 | 0.04(0.03-0.06) |  |
|  | Malawi | Stable (>=0.01%) | 1,003,800 | 1,003,800 | 100 | 19.21(10.24-30.59) |  | |  |  |  |  |
|  | Mali | Stable (>=0.01%) | 1,192,500 | 1,192,500 | 100 | 21.47(4.94-37.91) |  | |  |  |  |  |
|  | Mauritania | Unstable (<0.01%) | 205,100 | 400 | 0.2 | <0.001 |  | |  |  |  |  |
|  | Mauritania | Stable (>=0.01%) | 205,100 | 204,700 | 99.8 | 5.94(2.55-8.95) |  | |  |  |  |  |
|  | Mozambique | Stable (>=0.01%) | 1,759,100 | 1,759,100 | 100 | 29.49(9.2-53.81) |  | |  |  |  |  |
|  | Namibia | Unstable (<0.01%) | 79,100 | 1,300 | 1.64 | 0(0-0.01) |  | |  |  |  |  |
|  | Namibia | Stable (>=0.01%) | 79,100 | 77,800 | 98.36 | 0.95(0.04-2.21) |  | |  |  |  |  |
|  | Niger | Unstable (<0.01%) | 1,623,300 | 900 | 0.06 | <0.001 |  | |  |  |  |  |
|  | Niger | Stable (>=0.01%) | 1,623,300 | 1,622,400 | 99.94 | 24.33(11.5-34.9) |  | |  |  |  |  |
|  | Nigeria | Stable (>=0.01%) | 10,677,900 | 10,677,900 | 100 | 29.87(15.8-43.71) |  | |  |  |  |  |
|  | Republic of Congo | Stable (>=0.01%) | 257,000 | 257,000 | 100 | 20.42(12.68-33.03) |  | |  |  |  |  |
|  | Rwanda | Stable (>=0.01%) | 503,200 | 503,200 | 100 | 3.18(0.62-10.81) |  | |  |  |  |  |
| Sub-Saharan Africa | Senegal | Stable (>=0.01%) | 712,000 | 712,000 | 100 | 4.64(3.04-9.42) |  | |  |  |  |  |
|  | Sierra Leone | Stable (>=0.01%) | 328,800 | 328,800 | 100 | 39.19(22.74-46.04) |  | |  |  |  |  |
|  | Somalia | Stable (>=0.01%) | 966,400 | 966,400 | 100 | 6.65(2.85-11.53) | 960,300 | | 960,300 | 100 | 0.3(0.22-0.37) |  |
|  | South Africa | Unstable (<0.01%) | 286,900 | 36,500 | 12.72 | 0(0-0.01) |  | |  |  |  |  |
|  | South Africa | Stable (>=0.01%) | 286,900 | 250,400 | 87.28 | 0.11(0.02-0.24) |  | |  |  |  |  |
|  | South Sudan | Stable (>=0.01%) | 594,800 | 594,800 | 100 | 25.47(12.72-57.31) |  | |  |  |  |  |
|  | Tanzania | Stable (>=0.01%) | 3,193,700 | 3,193,700 | 100 | 8.13(2.92-16.1) |  | |  |  |  |  |
|  | Togo | Stable (>=0.01%) | 361,500 | 361,500 | 100 | 23.33(14.29-29.88) |  | |  |  |  |  |
|  | Uganda | Stable (>=0.01%) | 2,409,800 | 2,409,800 | 100 | 21.67(6.52-60.31) |  | |  |  |  |  |
|  | Zambia | Stable (>=0.01%) | 1,007,800 | 1,007,800 | 100 | 17.11(4.6-38.47) |  | |  |  |  |  |
|  | Zimbabwe | Stable (>=0.01%) | 643,700 | 643,700 | 100 | 4.07(2.9-6.65) |  | |  |  |  |  |
| Central & Southern Asia | Afghanistan | Unstable (<0.01%) | 2,157,000 | 1,722,800 | 79.87 | 0(0-0.01) | 2,158,900 | | 17,700 | 0.82 | 0(0-0.01) |  |
|  | Afghanistan | Stable (>=0.01%) | 2,157,000 | 434,200 | 20.13 | 0.06(0.01-0.35) | 2,158,900 | | 2,141,200 | 99.18 | 0.64(0.09-1.58) |  |
|  | Bangladesh | Unstable (<0.01%) | 1,339,500 | 1,061,200 | 79.22 | 0(0-0.01) | 1,266,000 | | 17,500 | 1.38 | 0.01(0-0.01) |  |
|  | Bangladesh | Stable (>=0.01%) | 1,339,500 | 278,300 | 20.78 | 0.19(0.01-1.1) | 1,266,000 | | 1,248,500 | 98.62 | 0.16(0.02-0.46) |  |
|  | Bhutan | Unstable (<0.01%) | 6,800 | 6,800 | 100 | <0.001 | 8,700 | | 1,000 | 11.49 | 0(0-0.01) |  |
|  | Bhutan | Stable (>=0.01%) |  |  |  |  | 8,700 | | 7,700 | 88.51 | 0.14(0.01-0.39) |  |
|  | India | Unstable (<0.01%) | 37,479,000 | 23,240,200 | 62.01 | 0(0-0.01) | 38,280,200 | | 1,082,700 | 2.83 | 0(0-0.01) |  |
|  | India | Stable (>=0.01%) | 37,479,000 | 14,238,800 | 37.99 | 0.23(0.01-1.61) | 38,280,200 | | 37,197,500 | 97.17 | 0.4(0.06-0.97) |  |
|  | Iran | Unstable (<0.01%) | 1,166,200 | 1,166,200 | 100 | <0.001 | 1,961,800 | | 1,655,300 | 84.38 | 0(0-0.01) |  |
|  | Iran | Stable (>=0.01%) |  |  |  |  | 1,961,800 | | 306,500 | 15.62 | 0.02(0.01-0.05) |  |
|  | Nepal | Unstable (<0.01%) | 446,000 | 446,000 | 100 | <0.001 | 598,200 | | 36,300 | 6.07 | 0(0-0.01) |  |
|  | Nepal | Stable (>=0.01%) |  |  |  |  | 598,200 | | 561,900 | 93.93 | 0.05(0.01-0.11) |  |
|  | Pakistan | Unstable (<0.01%) | 5,254,700 | 812,600 | 15.46 | 0(0-0.01) |  | |  |  |  |  |
|  | Pakistan | Stable (>=0.01%) | 5,254,700 | 4,442,100 | 84.54 | 0.26(0.01-0.98) | 10,257,700 | | 10,257,700 | 100 | 0.76(0.07-1.65) |  |
|  | Tajikistan | Unstable (<0.01%) |  |  |  |  | 33,000 | | 6,000 | 18.18 | 0(0-0.01) |  |
| Central & Southern Asia | Tajikistan | Stable (>=0.01%) |  |  |  |  | 33,000 | | 27,000 | 81.82 | 0.04(0.01-0.18) |  |
| Eastern & South-Eastern Asia | Brunei | Unstable (<0.01%) | 500 | 500 | 100 | <0.001 |  | |  |  |  |  |
|  | Cambodia | Unstable (<0.01%) | 589,200 | 362,600 | 61.54 | 0(0-0.01) |  | |  |  |  |  |
|  | Cambodia | Stable (>=0.01%) | 589,200 | 226,600 | 38.46 | 0.04(0.01-0.09) | 589,200 | | 589,200 | 100 | 0.65(0.12-1.75) |  |
|  | Indonesia | Unstable (<0.01%) | 5,807,500 | 4,000,800 | 68.89 | 0(0-0.01) | 6,718,800 | | 215,800 | 3.21 | 0(0-0.01) |  |
|  | Indonesia | Stable (>=0.01%) | 5,807,500 | 1,806,700 | 31.11 | 0.19(0.01-1.24) | 6,718,800 | | 6,503,000 | 96.79 | 0.55(0.03-2.24) |  |
|  | Laos | Unstable (<0.01%) | 280,200 | 186,400 | 66.52 | 0(0-0.01) |  | |  |  |  |  |
|  | Laos | Stable (>=0.01%) | 280,200 | 93,800 | 33.48 | 0.06(0.01-0.22) | 282,800 | | 282,800 | 100 | 0.3(0.05-0.75) |  |
|  | Malaysia | Unstable (<0.01%) |  |  |  |  | 79,300 | | 75,400 | 95.08 | 0(0-0.01) |  |
|  | Malaysia | Stable (>=0.01%) |  |  |  |  | 79,300 | | 3,900 | 4.92 | 0.02(0.01-0.03) |  |
|  | Myanmar | Unstable (<0.01%) | 1,608,000 | 274,400 | 17.06 | 0.01(0-0.01) | NA | | NA |  |  |  |
|  | Myanmar | Stable (>=0.01%) | 1,608,000 | 1,333,600 | 82.94 | 0.08(0.01-0.36) | 1,608,100 | | 1,608,100 | 100 | 0.91(0.38-1.81) |  |
|  | North Korea | Unstable (<0.01%) |  |  |  |  | 777,000 | | 7,300 | 0.94 | 0(0-0.01) |  |
|  | North Korea | Stable (>=0.01%) |  |  |  |  | 777,000 | | 769,700 | 99.06 | 0.07(0.01-0.16) |  |
|  | Papua New Guinea | Stable (>=0.01%) | 299,700 | 299,700 | 100 | 2.07(0.17-7.55) | 310,900 | | 307,000 | 98.75 | 2.95(0.68-5.55) |  |
|  | Papua New Guinea | Unstable (<0.01%) |  |  |  |  | 310,900 | | 3,900 | 1.25 | <0.001 |  |
|  | Philippines | Unstable (<0.01%) | 152,800 | 32,900 | 21.53 | 0(0-0.01) | 95,200 | | 3,300 | 3.47 | 0(0-0.01) |  |
|  | Philippines | Stable (>=0.01%) | 152,800 | 119,900 | 78.47 | 0.12(0.01-0.48) | 95,200 | | 91,900 | 96.53 | 0.3(0.04-0.82) |  |
|  | Solomon Islands | Stable (>=0.01%) | 19,500 | 19,500 | 100 | 1.13(0.06-6.77) | 19,500 | | 19,500 | 100 | 4.84(2.67-7.62) |  |
|  | South Korea | Unstable (<0.01%) |  |  |  |  | 702,700 | | 140,100 | 19.94 | 0(0-0.01) |  |
|  | South Korea | Stable (>=0.01%) |  |  |  |  | 702,700 | | 562,600 | 80.06 | 0.02(0.01-0.04) |  |
|  | Thailand | Unstable (<0.01%) |  |  |  |  | 434,100 | | 88,700 | 20.43 | 0(0-0.01) |  |
|  | Thailand | Stable (>=0.01%) |  |  |  |  | 434,100 | | 345,400 | 79.57 | 0.18(0.01-0.57) |  |
|  | Vanuatu | Unstable (<0.01%) | 8,100 | 7,400 | 91.36 | 0(0-0.01) |  | |  |  |  |  |
|  | Vanuatu | Stable (>=0.01%) | 8,100 | 700 | 8.64 | 0.03(0.02-0.05) | 8,200 | | 8,200 | 100 | 0.52(0.1-1.16) |  |
|  | Vietnam | Unstable (<0.01%) | 1,324,500 | 1,256,800 | 94.89 | 0(0-0.01) | 1,274,400 | | 86,400 | 6.78 | 0(0-0.01) |  |
|  | Vietnam | Stable (>=0.01%) | 1,324,500 | 67,700 | 5.11 | 0.03(0.01-0.15) | 1,274,400 | | 1,188,000 | 93.22 | 0.07(0.01-0.23) |  |
| Latin America & the Caribbean | Argentina | Unstable (<0.01%) | 10,000 | 10,000 | 100 | <0.001 | 24,900 | | 10,500 | 42.17 | <0.001 |  |
|  | Argentina | Stable (>=0.01%) | NA | NA |  |  | 24,900 | | 14,400 | 57.83 | 0.02(0.01-0.04) |  |
|  | Belize | Unstable (<0.01%) | NA | NA |  |  | 7,900 | | 3,100 | 39.24 | <0.001 |  |
|  | Belize | Stable (>=0.01%) | NA | NA |  |  | 7,900 | | 4,800 | 60.76 | 0.02(0.01-0.02) |  |
|  | Bolivia | Unstable (<0.01%) | 12,300 | 12,300 | 100 | 0(0-0.01) | 331,700 | | 13,200 | 3.98 | 0(0-0.01) |  |
|  | Bolivia | Stable (>=0.01%) |  |  |  |  | 331,700 | | 318,500 | 96.02 | 0.5(0.02-1.94) |  |
|  | Brazil | Unstable (<0.01%) | 2,156,600 | 1,820,900 | 84.43 | <0.001 | 3,186,100 | | 900,300 | 28.26 | 0(0-0.01) |  |
|  | Brazil | Stable (>=0.01%) | 2,156,600 | 335,700 | 15.57 | 0.29(0.01-1.09) | 3,186,100 | | 2,285,800 | 71.74 | 0.25(0.01-1.43) |  |
|  | Colombia | Unstable (<0.01%) | 862,900 | 233,000 | 27 | 0(0-0.01) | 907,900 | | 51,800 | 5.71 | 0.01(0-0.01) |  |
|  | Colombia | Stable (>=0.01%) | 862,900 | 629,900 | 73 | 0.38(0.01-4.36) | 907,900 | | 856,100 | 94.29 | 0.32(0.01-1.54) |  |
|  | Costa Rica | Unstable (<0.01%) | 98,400 | 98,400 | 100 | <0.001 | 9,300 | | 7,600 | 81.72 | 0(0-0.01) |  |
|  | Costa Rica | Stable (>=0.01%) |  |  |  |  | 9,300 | | 1,700 | 18.28 | 0.02(0.02-0.02) |  |
|  | Dominican Republic | Unstable (<0.01%) | 227,300 | 149,900 | 65.95 | 0(0-0.01) |  | |  |  |  |  |
|  | Dominican Republic | Stable (>=0.01%) | 227,300 | 77,400 | 34.05 | 0.03(0.01-0.16) |  | |  |  |  |  |
|  | Ecuador | Unstable (<0.01%) | 341,300 | 43,800 | 12.83 | 0(0-0.01) | 445,600 | | 66,300 | 14.88 | 0(0-0.01) |  |
|  | Ecuador | Stable (>=0.01%) | 341,300 | 297,500 | 87.17 | 0.11(0.01-0.4) | 445,600 | | 379,300 | 85.12 | 0.18(0.01-0.71) |  |
|  | French Guiana | Stable (>=0.01%) | 11,800 | 11,800 | 100 | 0.69(0.32-1.07) | 11,800 | | 11,800 | 100 | 0.29(0.18-0.44) |  |
|  | Guatemala | Unstable (<0.01%) | 4,300 | 4,300 | 100 | <0.001 | 411,800 | | 104,000 | 25.25 | 0(0-0.01) |  |
| Latin America & the Caribbean | Guatemala | Stable (>=0.01%) |  |  |  |  | 411,800 | | 307,800 | 74.75 | 0.13(0.01-0.36) |  |
|  | Guyana | Stable (>=0.01%) | 20,900 | 20,900 | 100 | 1.02(0.65-2.15) | 20,900 | | 20,900 | 100 | 1.52(0.78-3.13) |  |
|  | Haiti | Unstable (<0.01%) | 423,100 | 125,600 | 29.69 | 0(0-0.01) |  | |  |  |  |  |
|  | Haiti | Stable (>=0.01%) | 423,100 | 297,500 | 70.31 | 0.21(0.01-1.77) |  | |  |  |  |  |
|  | Honduras | Unstable (<0.01%) | 243,100 | 237,500 | 97.7 | 0(0-0.01) | 245,000 | | 54,000 | 22.04 | 0(0-0.01) |  |
|  | Honduras | Stable (>=0.01%) | 243,100 | 5,600 | 2.3 | 0.08(0.01-0.23) | 245,000 | | 191,000 | 77.96 | 0.11(0.01-0.35) |  |
|  | Mexico | Unstable (<0.01%) | NA | NA |  |  | 765,900 | | 110,200 | 14.39 | <0.001 |  |
|  | Mexico | Stable (>=0.01%) | NA | NA |  |  | 765,900 | | 655,700 | 85.61 | 0.04(0.01-0.09) |  |
|  | Nicaragua | Unstable (<0.01%) | 170,100 | 67,100 | 39.45 | 0(0-0.01) | 173,200 | | 17,600 | 10.16 | 0(0-0.01) |  |
|  | Nicaragua | Stable (>=0.01%) | 170,100 | 103,000 | 60.55 | 0.15(0.01-0.74) | 173,200 | | 155,600 | 89.84 | 0.32(0.01-1.68) |  |
|  | Panama | Unstable (<0.01%) | NA | NA |  |  | 37,400 | | 12,800 | 34.22 | 0(0-0.01) |  |
|  | Panama | Stable (>=0.01%) | NA | NA |  |  | 37,400 | | 24,600 | 65.78 | 0.7(0.02-1.88) |  |
|  | Paraguay | Unstable (<0.01%) | 5,300 | 5,300 | 100 | <0.001 | 12,700 | | 12,000 | 94.49 | 0(0-0.01) |  |
|  | Paraguay | Stable (>=0.01%) | NA | NA |  |  | 12,700 | | 700 | 5.51 | 0.02(0.02-0.02) |  |
|  | Peru | Unstable (<0.01%) | 716,800 | 671,600 | 93.69 | <0.001 | 364,800 | | 43,200 | 11.84 | 0(0-0.01) |  |
|  | Peru | Stable (>=0.01%) | 716,800 | 45,200 | 6.31 | 0.65(0.02-1.81) | 364,800 | | 321,600 | 88.16 | 0.26(0.01-1.27) |  |
|  | Suriname | Stable (>=0.01%) | NA | NA |  |  | 17,400 | | 17,400 | 100 | 0.17(0.09-0.3) |  |
|  | Venezuela | Unstable (<0.01%) | 674,800 | 423,600 | 62.77 | 0(0-0.01) | 675,200 | | 59,900 | 8.87 | 0(0-0.01) |  |
|  | Venezuela | Stable (>=0.01%) | 674,800 | 251,200 | 37.23 | 1.29(0.01-11.04) | 675,200 | | 615,300 | 91.13 | 0.73(0.04-3.48) |  |
