# Supplementary material for: Global estimates of pregnancies at risk of Plasmodium falciparum and Plasmodium vivax infection in 2020 and changes in risk patterns since 2000
Source: PLOS Glob Public Health. 2022 Nov 9;2(11):e0001061. doi: 10.1371/journal.pgph.0001061 (PMC10022219; doi:10.1371/journal.pgph.0001061)
Supplement: S3 Table — (DOCX) [file pgph.0001061.s003.docx]

| **SDG region** | **Pregnancies at risk of**  **P. falciparum and P. vivax (%)** | **% of global total** |
| --- | --- | --- |
| **Northern Africa & Western Asia** | 2,615,500 | 4.0 |
| **Sub-Saharan Africa** | 6,978,800 | 10.8 |
| **Central & Southern Asia** | 41,521,800 | 64.2 |
| **Eastern & South-Eastern Asia** | 9,587,400 | 14.8 |
| **Latin America & the Caribbean** | 3,927,600 | 6.1 |
| **Global Total** | 64,631,100 |  |

# *S3 Table*: Pregnancies at risk of *Plasmodium falciparum* and *Plasmodium vivax* in 2020 by regions of the Sustainable Development Goals
