# Supplementary material for: Global estimates of pregnancies at risk of Plasmodium falciparum and Plasmodium vivax infection in 2020 and changes in risk patterns since 2000
Source: PLOS Glob Public Health. 2022 Nov 9;2(11):e0001061. doi: 10.1371/journal.pgph.0001061 (PMC10022219; doi:10.1371/journal.pgph.0001061)
Supplement: S4 Table — (DOCX) [file pgph.0001061.s004.docx]

|  | **Year** | **Northern Africa & Western Asia** | | | **Sub-Saharan Africa** | | | **Central & Southern Asia** | | | **Eastern & South-Eastern Asia** | | | **Latin America & the Caribbean** | | | **Global Total** | |
| --- | --- | --- | --- | --- | --- | --- | --- | --- | --- | --- | --- | --- | --- | --- | --- | --- | --- | --- |
|  |  | **Total Pregnancies** | **Pregnancies at risk of malaria, n (%)** | **Total Pregnancies** | | **Pregnancies at risk of malaria, n (%)** | **Total Pregnancies** | | **Pregnancies at risk of malaria, n (%)** | **Total Pregnancies** | | **Pregnancies at risk of malaria, n (%)** | **Total Pregnancies** | | **Pregnancies at risk of malaria, n (%)** | **Total Pregnancies** | | **Pregnancies at risk of malaria, n (%)** |
| ***P.falciparum*** | 2000 | 7.8 | 5.2(66.67) | 39.2 | | 37.3(95.15) | 65.7 | | 65.2(99.24) | 55.9 | | 19.8(35.42) | 17.8 | | 13.3(74.72) | 186.4 | | 140.8(75.54) |
|  | 2005 | 8.1 | 3.8(46.91) | 43.4 | | 41.4(95.39) | 65.1 | | 60.1(92.32) | 56.3 | | 19.9(35.35) | 17.1 | | 12.4(72.51) | 190 | | 137.7(72.47) |
|  | 2010 | 8.6 | 3.9(45.35) | 47.4 | | 45.2(95.36) | 62.3 | | 55.3(88.76) | 57 | | 18.3(32.11) | 16.8 | | 7.6(45.24) | 192.1 | | 130.4(67.88) |
|  | 2015 | 8.8 | 4(45.45) | 50.7 | | 48.6(95.86) | 62.4 | | 53.9(86.38) | 54.9 | | 15.5(28.23) | 16.6 | | 6.6(39.76) | 193.4 | | 128.8(66.6) |
|  | 2017 | 8.9 | 4.1(46.07) | 52.6 | | 50.5(96.01) | 61.6 | | 52.2(84.74) | 53 | | 10.2(19.25) | 16.3 | | 6.1(37.42) | 192.3 | | 123.1(64.01) |
|  | 2020 | 8.9 | 4.1(46.07) | 54.5 | | 52.4(96.15) | 61.2 | | 47.2(77.12) | 51.1 | | 10.1(19.77) | 16 | | 6.3(39.38) | 191.6 | | 120.4(62.84) |
| ***P.vivax*** | 2000 | 7.8 | 5.5(70.51) | 6 | | 5.9(98.33) | 64.6 | | 64.5(99.85) | 56.8 | | 55(96.83) | 17.3 | | 14.9(86.13) | 152.6 | | 145.9(95.61) |
|  | 2005 | 8.1 | 5.8(71.6) | 6.3 | | 5.7(90.48) | 63.9 | | 61(95.46) | 57 | | 54.9(96.32) | 16.6 | | 13.2(79.52) | 152 | | 140.7(92.57) |
|  | 2010 | 8.6 | 3.4(39.53) | 6.8 | | 6.1(89.71) | 61 | | 57.1(93.61) | 57.7 | | 44.2(76.6) | 16.4 | | 9.1(55.49) | 150.5 | | 120(79.73) |
|  | 2015 | 8.8 | 3.4(38.64) | 7.3 | | 7.2(98.63) | 61 | | 55.4(90.82) | 55.6 | | 17.8(32.01) | 16.1 | | 7.1(44.1) | 148.9 | | 90.9(61.05) |
|  | 2017 | 8.8 | 3.9(44.32) | 7.6 | | 7.4(97.37) | 60.3 | | 54.8(90.88) | 53.7 | | 11.7(21.79) | 15.8 | | 7.1(44.94) | 146.3 | | 85(58.1) |
|  | 2020 | 8.8 | 3.1(35.23) | 7 | | 7(100) | 59.9 | | 54.6(91.15) | 51.8 | | 12.9(24.9) | 15.6 | | 7.6(48.72) | 143.1 | | 85.2(59.54) |

# *S4 Table:* Total number of pregnancies (millions) and pregnancies at risk of *P.falciparum* or *P.vivax* by SDG, 2000 to 2020
