# Supplementary figures and images for: Global estimates of pregnancies at risk of Plasmodium falciparum and Plasmodium vivax infection in 2020 and changes in risk patterns since 2000
Source: PLOS Glob Public Health. 2022 Nov 9;2(11):e0001061. doi: 10.1371/journal.pgph.0001061 (PMC10022219; doi:10.1371/journal.pgph.0001061)

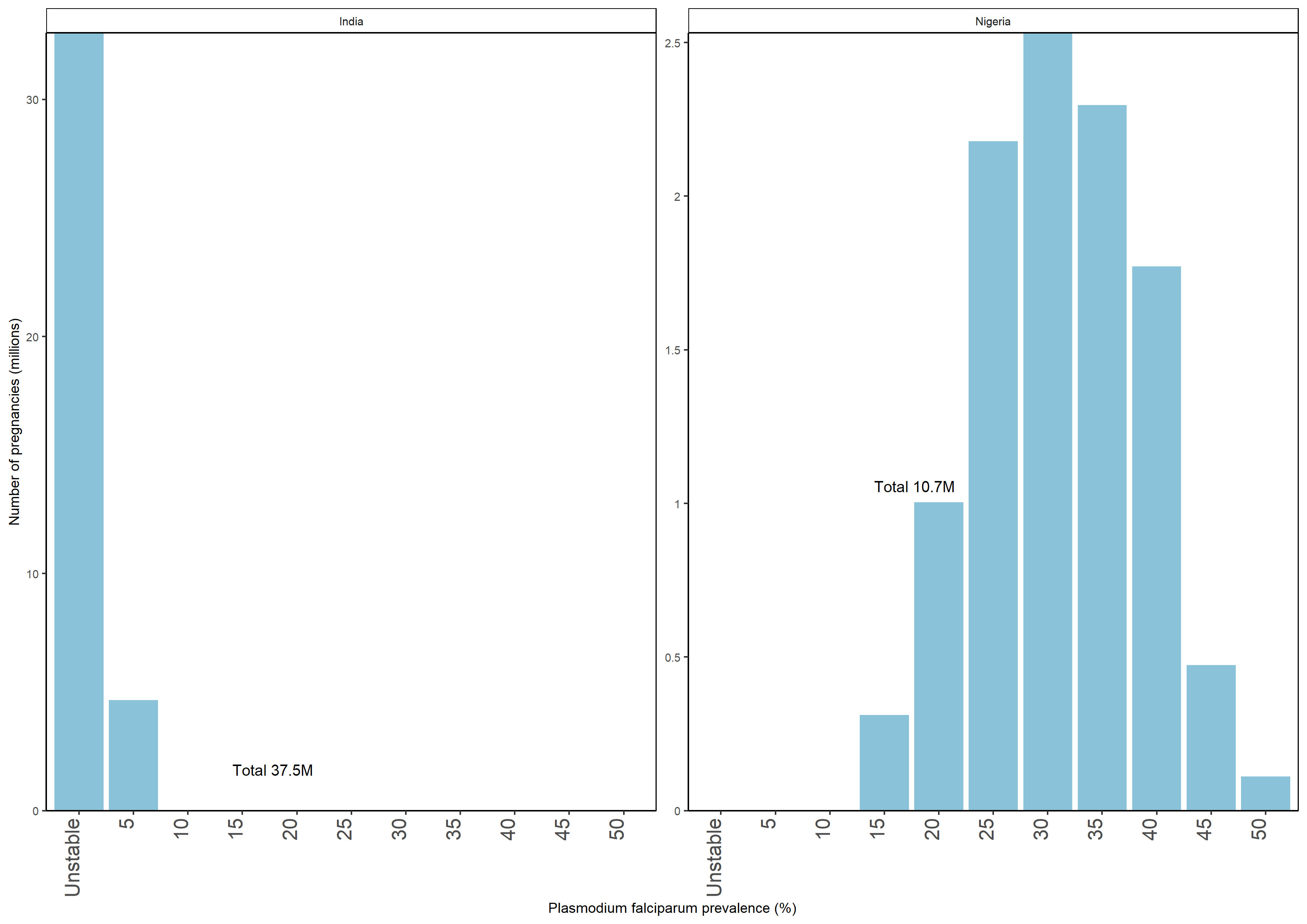

Supplement: S1 Fig — (TIFF) [file pgph.0001061.s005.tiff]

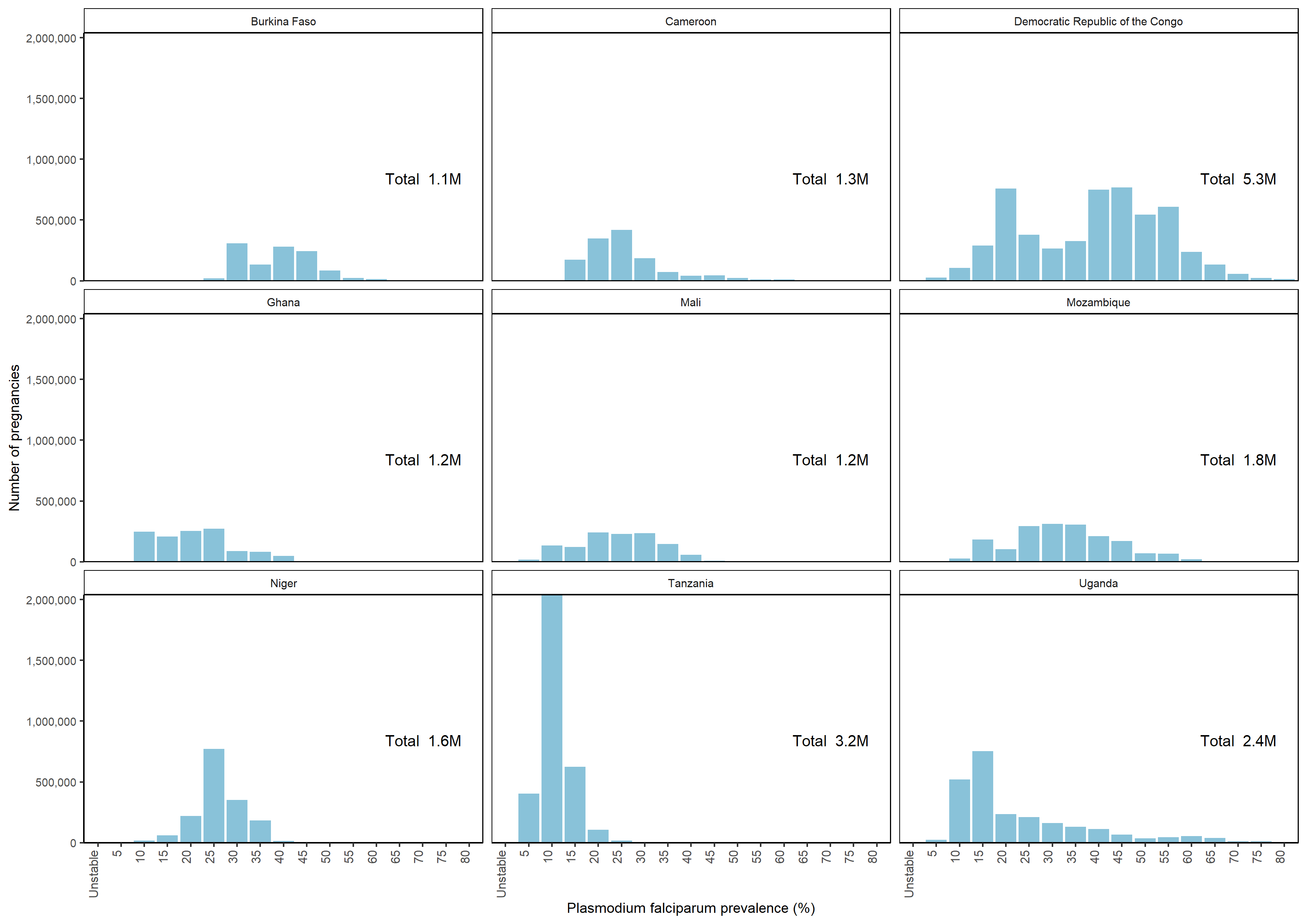

Supplement: S2 Fig — (TIFF) [file pgph.0001061.s006.tiff]

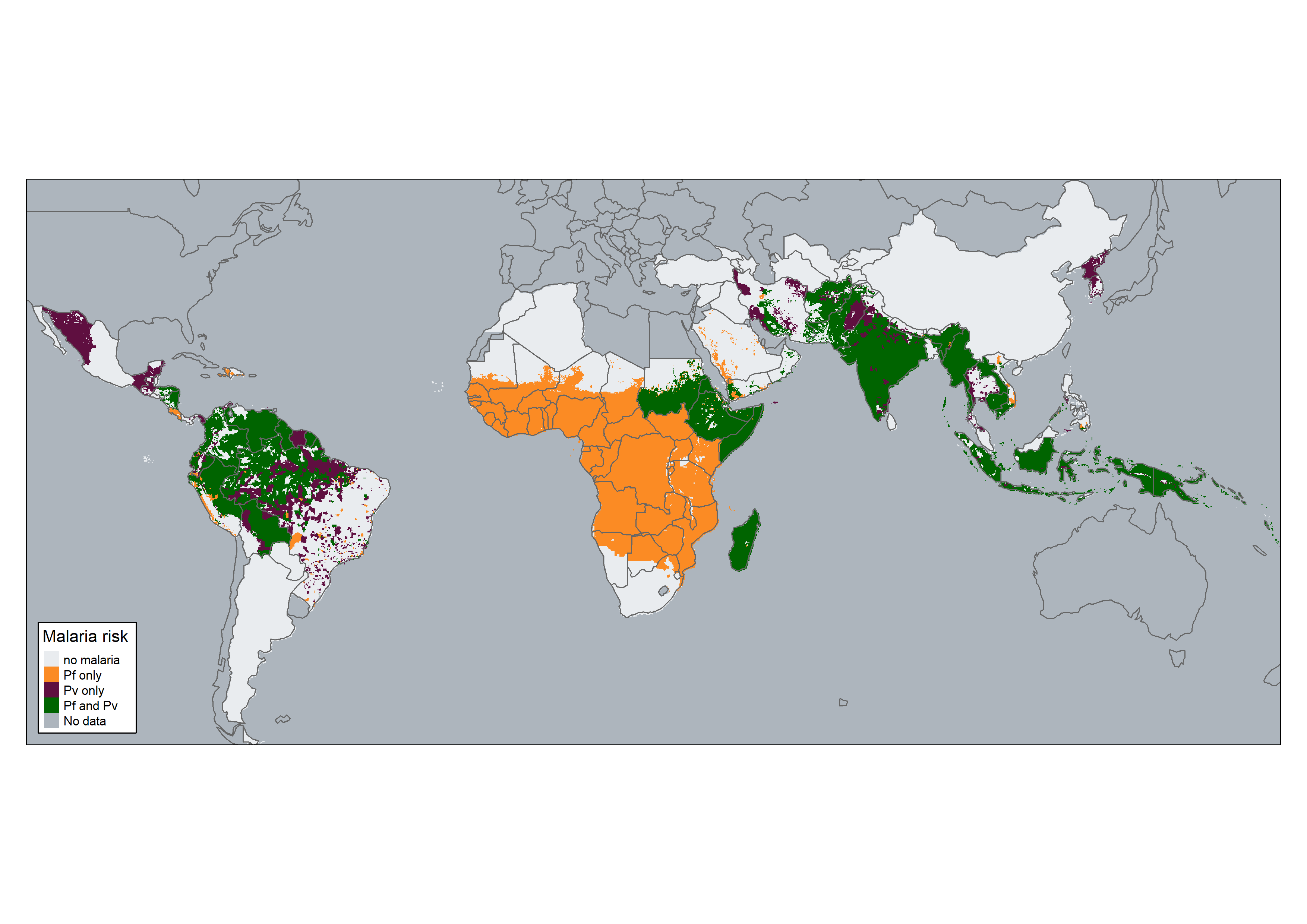

Supplement: S3 Fig — Note: Map made with Natural Earth. Free vector and raster map data @ naturalearthdata.com. (TIFF) [file pgph.0001061.s007.tiff]

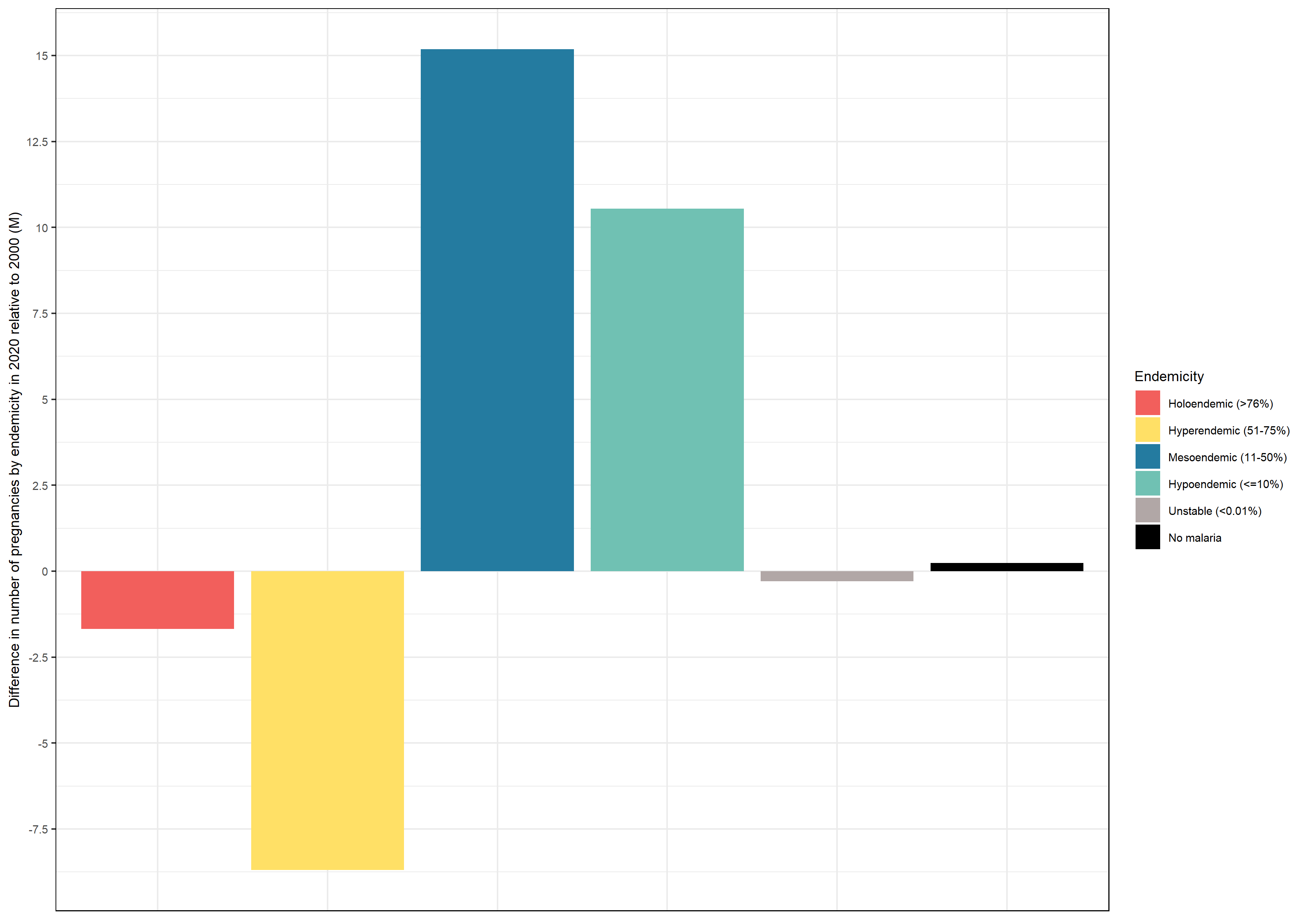

Supplement: S4 Fig — (TIFF) [file pgph.0001061.s008.tiff]

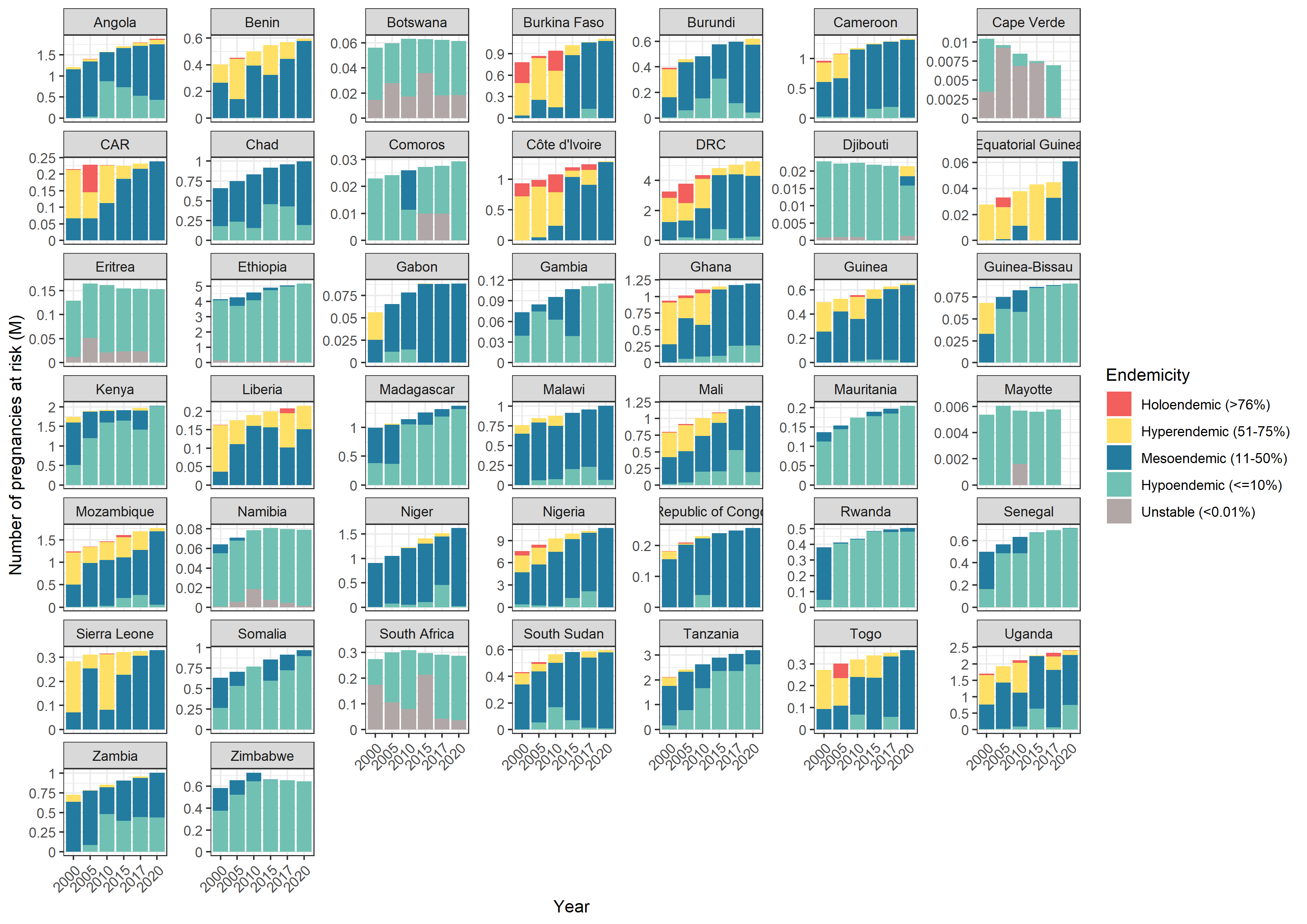

Supplement: S5 Fig — (TIFF) [file pgph.0001061.s009.tiff]

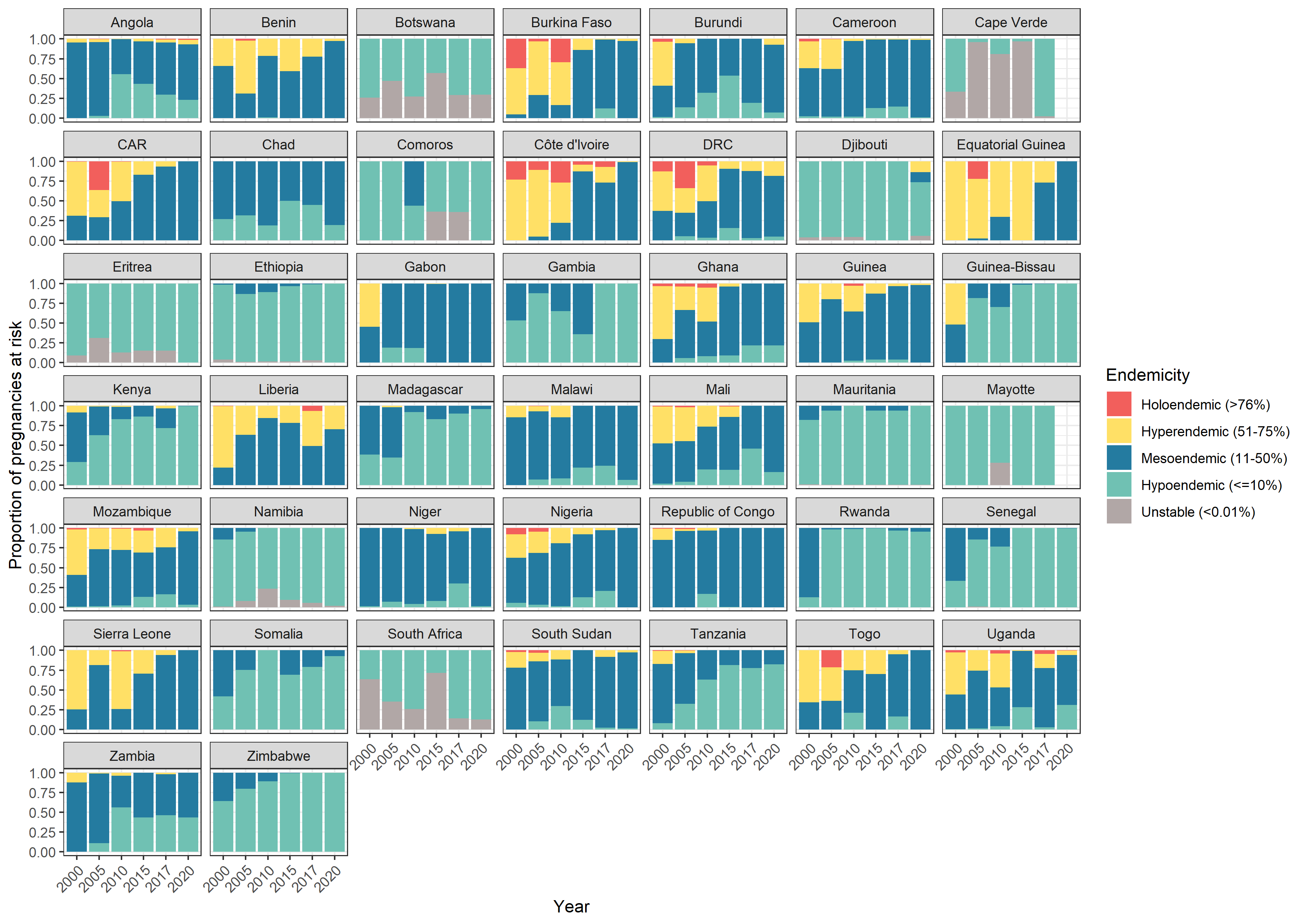

Supplement: S6 Fig — (TIFF) [file pgph.0001061.s010.tiff]

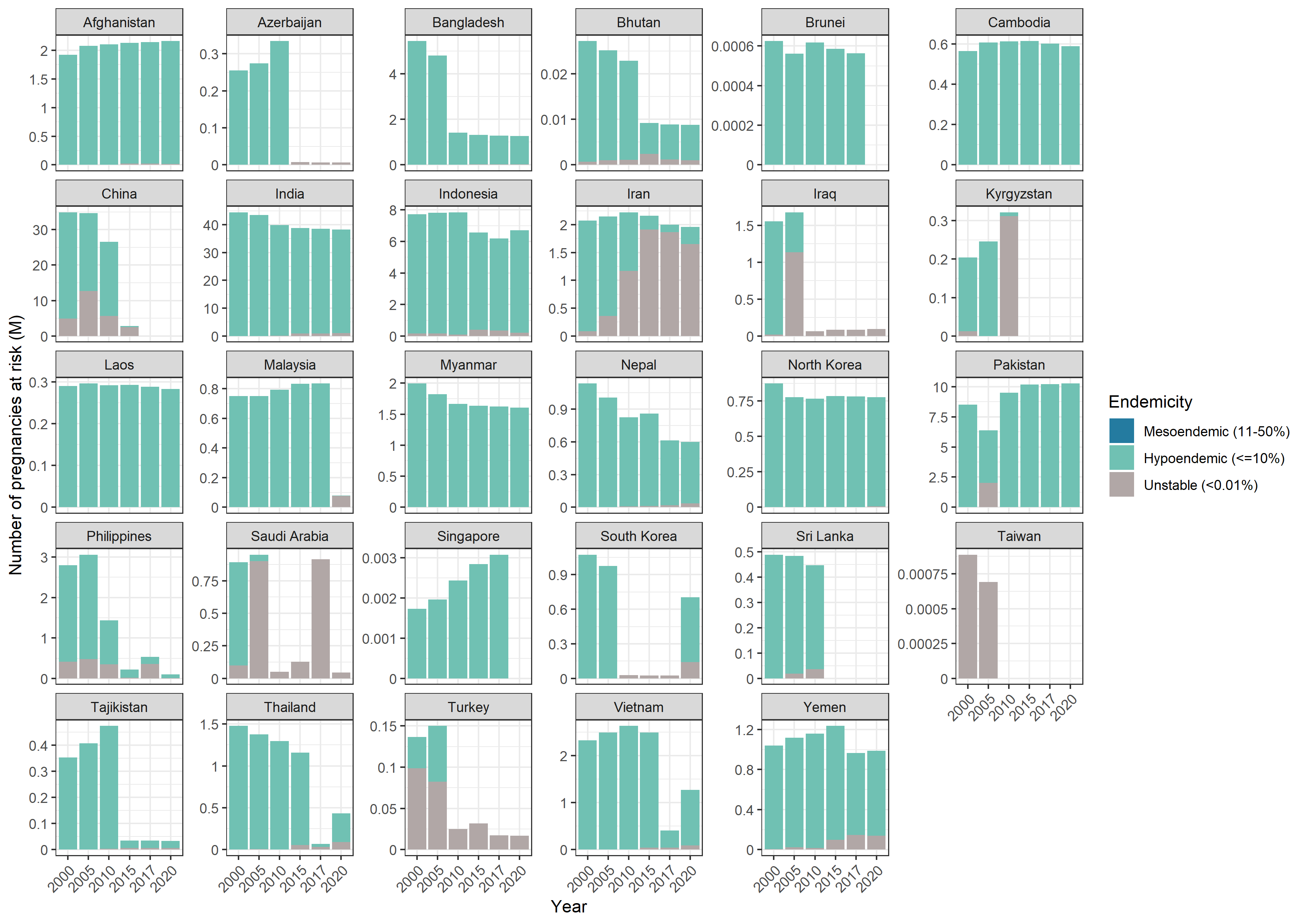

Supplement: S7 Fig — (TIFF) [file pgph.0001061.s011.tiff]

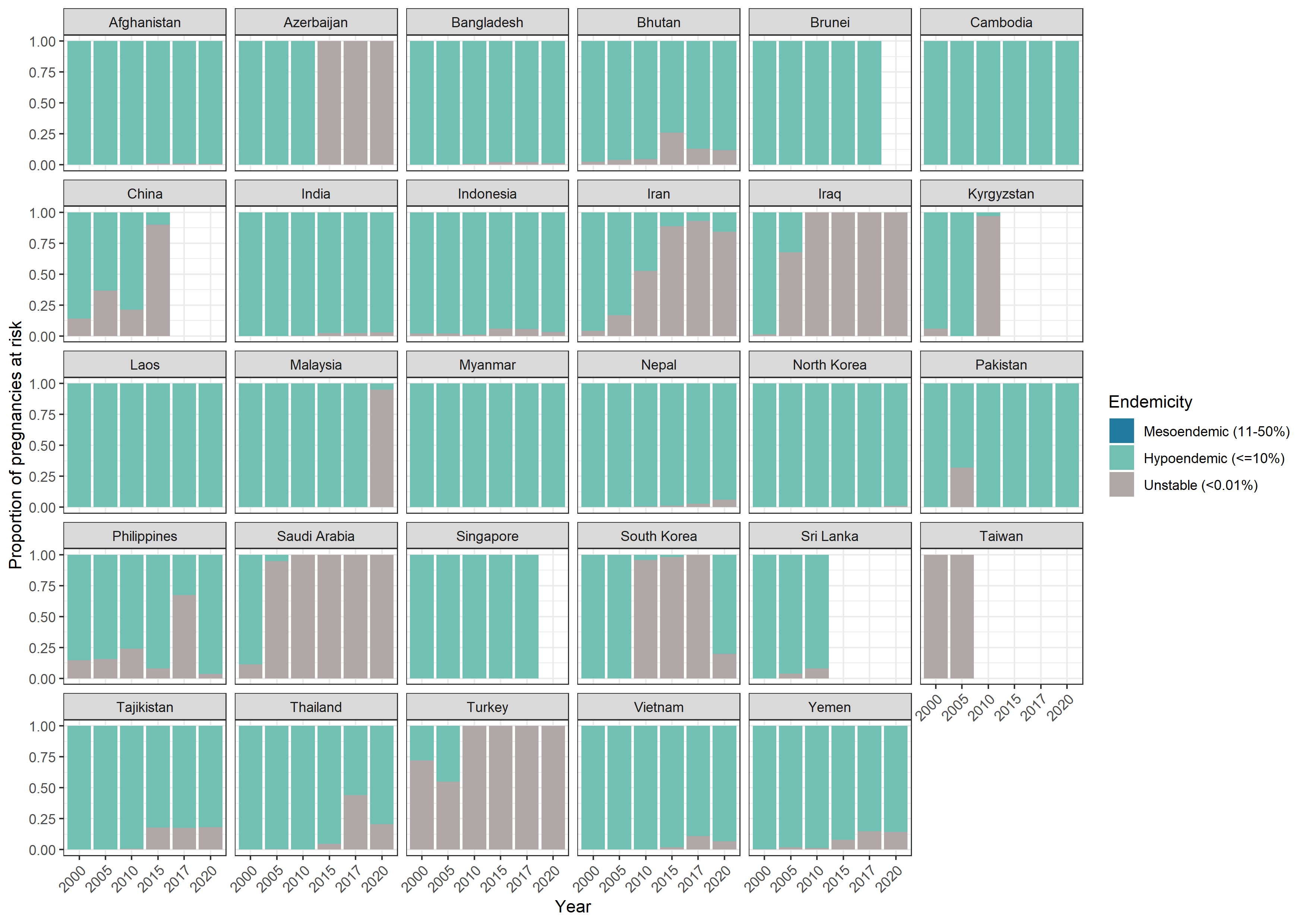

Supplement: S8 Fig — (TIFF) [file pgph.0001061.s012.tiff]

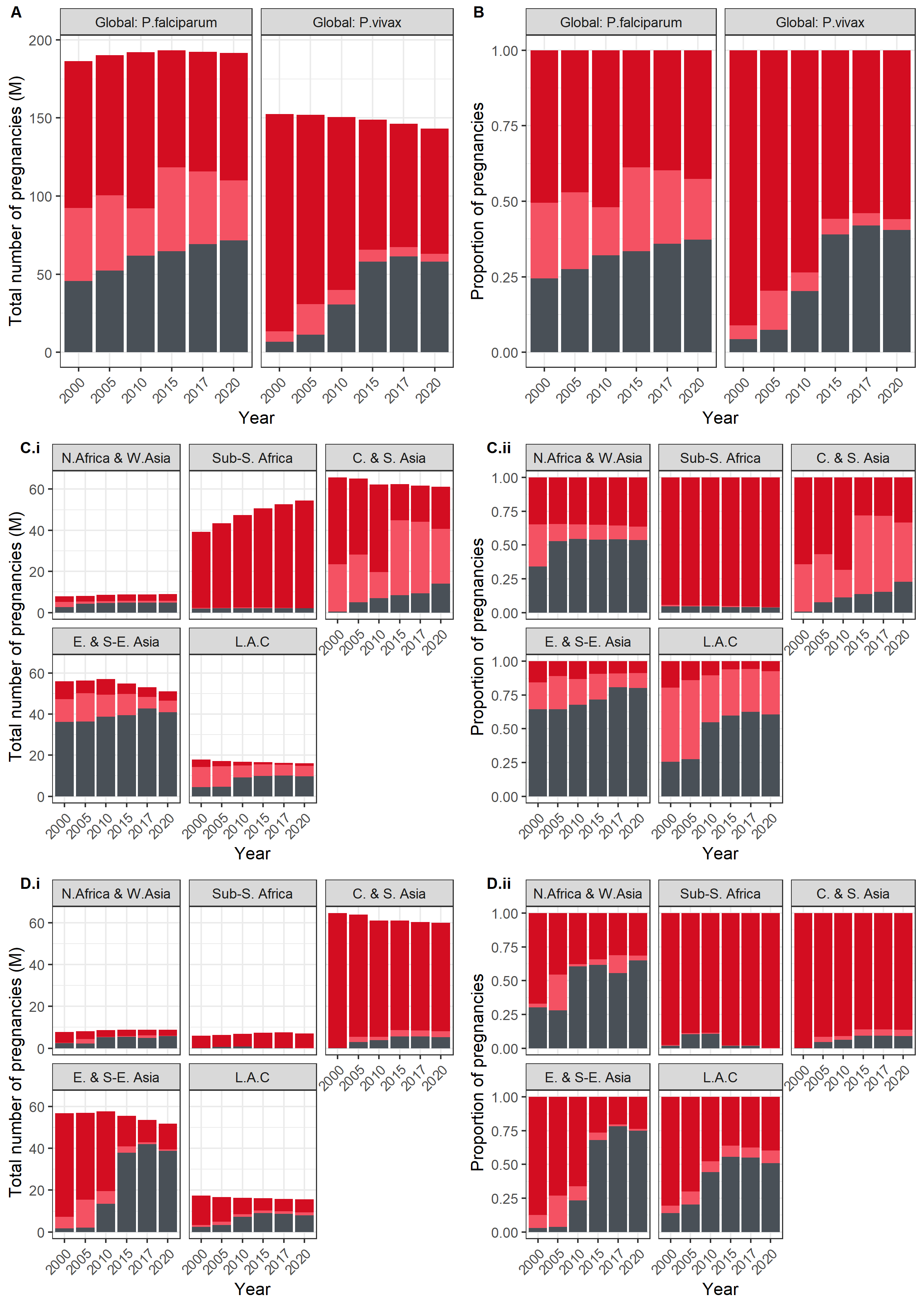

Supplement: S9 Fig — Number (A) and proportion (B) of pregnancies in countries of Plasmodium falciparum and Plasmodium vivax transmission globally from 2000 to 2020 disaggregated by risk of malaria or no risk of malaria, and number (C.i) and proportion (C.ii) of pregnancies by risk of Plasmodium falciparum and Plasmodium vivax (D.i and D.ii) from 2000 to 2020 by geographic region. (TIFF) [file pgph.0001061.s013.tiff]

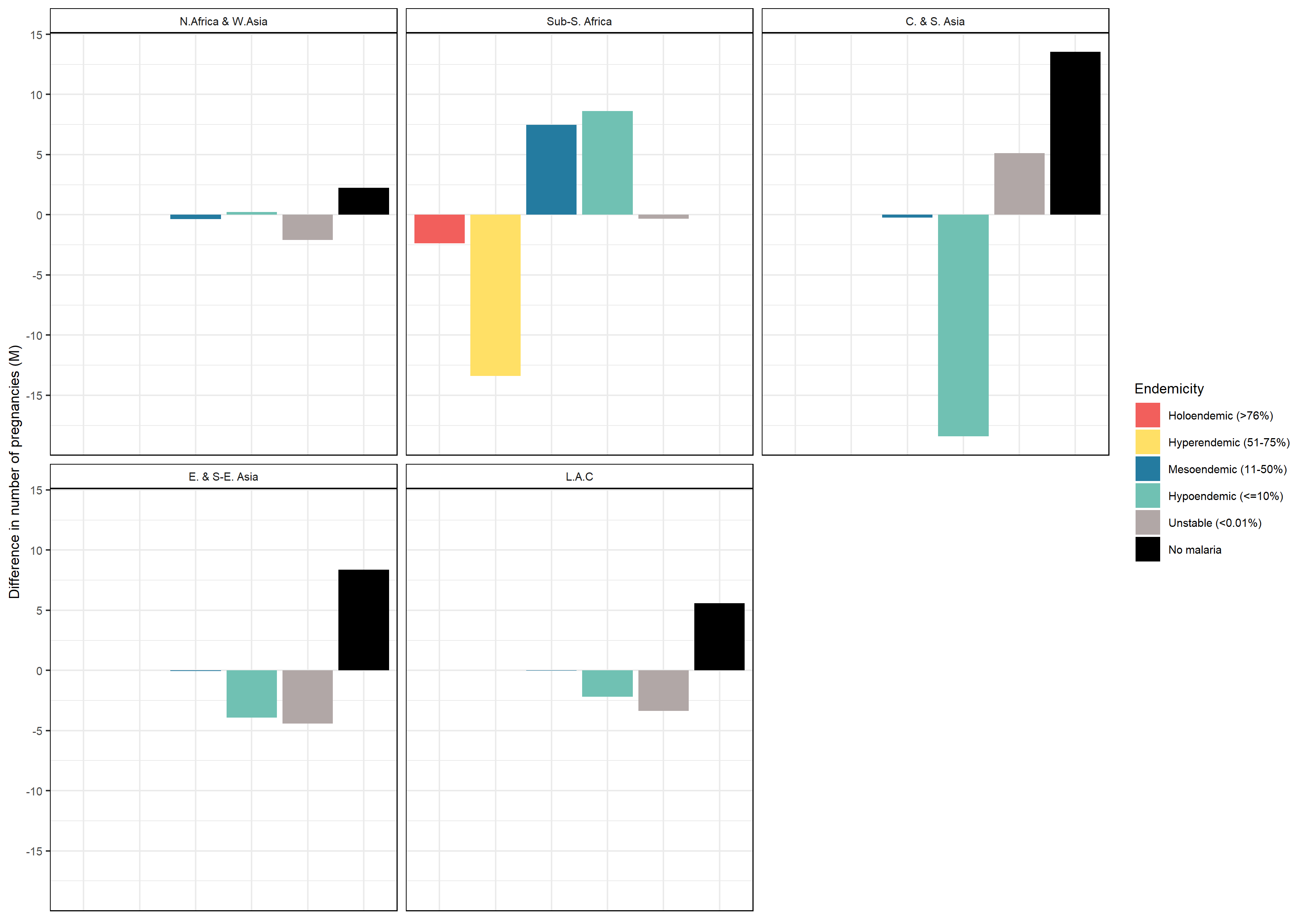

Supplement: S10 Fig — (TIFF) [file pgph.0001061.s014.tiff]

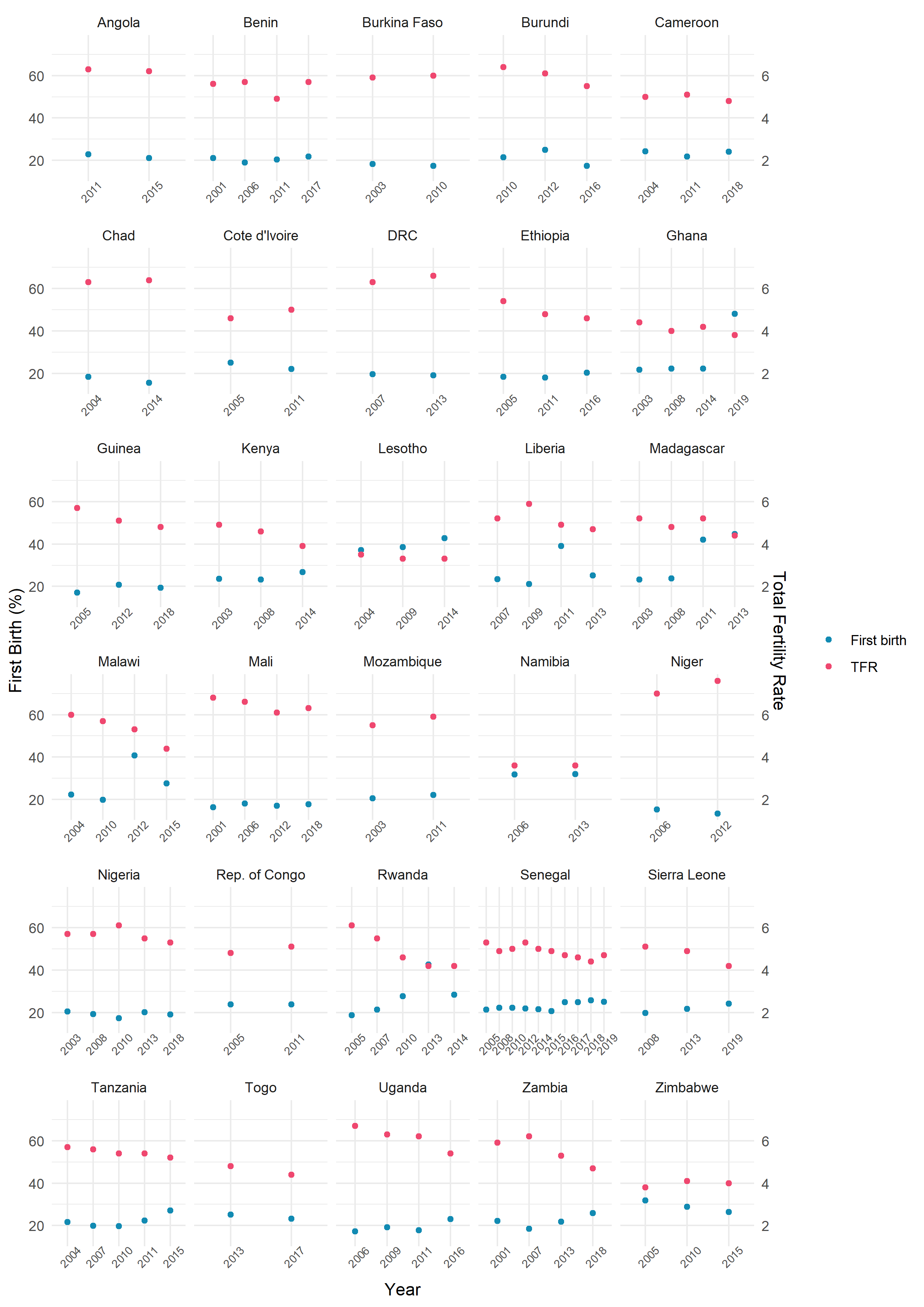

Supplement: S11 Fig — (TIFF) [file pgph.0001061.s015.tiff]
